# Total Flavonoids of Litchi Seed Attenuate Prostate Cancer Progression *Via* Inhibiting AKT/mTOR and NF-kB Signaling Pathways

**DOI:** 10.3389/fphar.2021.758219

**Published:** 2021-09-23

**Authors:** Ming Chang, Dan Zhu, Yanjiang Chen, Weiquan Zhang, Xi Liu, Xiao-Lan Li, Zhiping Cheng, Zhiheng Su, Jian Zhang, Yi Lu, Hongwei Guo

**Affiliations:** ^1^ Key Laboratory of Longevity and Aging-Related Diseases of Chinese Ministry of Education, Center for Translational Medicine, Guangxi Medical University, Nanning, China; ^2^ School of Medicine, Southern University of Science and Technology, Shenzhen, China; ^3^ Guangdong Provincial Key Laboratory of Cell Microenvironment and Disease Research, Shenzhen, China; ^4^ Guangxi Key Laboratory of Bioactive Molecules Research and Evaluation, College of Pharmacy, Guangxi Medical University, Nanning, China; ^5^ Department of Surgery, University of Melbourne, Parkville, VIC, Australia

**Keywords:** total flavonoids of litchi seed, prostate cancer, apoptosis, proliferation, metastasis, Akt

## Abstract

Litchi seeds have been traditionally used in Chinese herbal formula for urologic neoplasms including prostate cancer (PCa). However, the effective components of Litchi seeds and the mechanisms of their actions on PCa cell growth and metastasis remain unclear. In this study, we investigated the effects and molecular mechanisms of the Total Flavonoid of Litchi Seed (TFLS) in PCa PC3 and DU145 cell lines. We found that TFLS significantly inhibited the PCa cell proliferation, induced apoptosis, and prevented cell migration and invasion. Furthermore, we observed that TFLS upregulated the expression of epithelial biomarker E-cadherin and downregulated mesenchymal biomarker Vimentin. TFLS also increased the expression of cleaved-PRAP and Bax, and decreased the expression of Bcl-2 in both PC3 and DU145 cells. Besides, TFLS inhibited AKT signaling pathway by reducing the phosphorylation of AKT and activities of downstream signal transducers including mTOR, IκBα and NF-kB. Finally, TFLS treated mice exhibited a significant decrease in tumor size without toxicity in major organs *in vivo*. These results indicated that TFLS could suppress PCa cell growth *in vivo* and inhibit PCa cell proliferation and metastasis *in vitro* through induction of apoptosis and phenotypic reversal of EMT, which may be achieved by inhibiting the AKT/mTOR and NF-κB signaling pathways. Taken together, our data provide new insights into the role of TFLS as a novel potent anti-cancer agent for the treatment of PCa.

## Introduction

Prostate cancer is one of the most commonly diagnosed malignant tumors in men with high mortality, which causes a severe global health burden (Oliver et al., 1998; Karimi Roshan et al., 2019; Siegel et al., 2019). In China, the incidence of PCa has increased rapidly due to population aging, and PCa has the tendency to become the most common malignant tumor in the near future (Chen W. et al., 2016). Although patients with local PCa have a relatively high 5-year survival rate, most of the patients are diagnosed with bone metastasis, which almost inevitably causes deaths (Nieder et al., 2010). Currently, standard treatments for PCa include androgen antagonists, chemotherapy, radiotherapy and immunotherapy. These treatments are often accompanied with side effects that affect the general health and quality of life of patients (Farolfi et al., 2019). Hence, there is an urgent need to develop novel therapeutics that could effectively targeting PCa with low toxicity and few side effects.

In the past few years, natural herbal extracts have been of great research interests due to their drug specificity and limited side effects (Sharifi-Rad et al., 2019). As a supplement to conventional anticancer treatments, herbal extracts can alleviate clinical symptoms, prolong patient's survival and improve the quality of life of patients (Koga et al., 2017). Herbal extracts can also attenuate chemotherapy-related side effects, including nausea and vomiting. Therefore, herbal extracts could be promising therapeutic agents (Inoue et al., 2017).

*Litchi chinensis Sonn* (Litchi) belongs to the *Sapindaceae* family. Its seeds have been traditionally used in China for treating male urogenital diseases, including prostate cancer, prostatitis, benign prostatic hyperplasia and orchitis etc. (Cao et al., 2020). Accumulating pharmacological evidence suggested that the extracts of Litchi seeds have anticancer effects and can inhibit metastatic tumor growth (Hsu et al., 2012; Emanuele et al., 2018). We previously showed that the n-butyl alcohol extract of Litchi seeds demonstrated anti-prostate cancer properties both *in vitro* and *in vivo*, and flavonoid compounds were the most abundant chemical components (Guo et al., 2017). In this study, we examined the effects and mechanisms of Total Flavonoids of Litchi Seed (TFLS) on PCa. This study will provide the rationale for the clinical application of Litchi seeds for the treatment of PCa.

## Methods

### Material and Chemical Reagents

The fetal bovine serum (FBS) was purchased from Gibco (Grand Island, NY, United States). BCA protein assay kit (No. P0011) and One Step TUNEL Apoptosis Assay Kit (No. C1089) were purchased from Beyotime (Shanghai, China). Transwell assay inserts (No. 354483) were purchased from Corning (Corning New York, NY, United States). Enhanced Chemiluminescence (ECL) was purchased from Thermofisher (Waltham, Massachusetts, United States). 1% crystal violet solution (No. V5265) was purchased from Sigma (St. Louis, MO, United States). Anti-rabbit IgG, HRP-linked Antibody, primary antibodies against E-cadherin, Vimentin, p-AMPKα, p-p44/42 MAPK, p-MEK1/2, p-AKT, AKT, p-mTOR, mTOR, p-IκBα, IκBα, p-NF-κB p65, NF-κB p65 and GAPDH were purchased from Cell Signaling Technology (Danvers, MA, United States). Primary antibody against Wnt 3a was purchased from Abcam (Cambridge, United Kingdom). The catalogue numbers of antibodies were shown in Supplementary Table S1. FITC Annexin V Apoptosis Detection Kit (No. 556547) and DNA Reagent Kit (No. 340242) were purchased from BD Biosciences (San Diego, CA, United States). Cell Titer 96^®^ AQueous One Solution Cell Proliferation Assay (MTS, No. G3581) was purchased from Promega (Madison, WI, United States). The macroporous resin (No. M0032) was purchased from Solarbio Life Science (Beijing, China).

### Cell Lines and Culture Conditions

PC3, DU145 and PC3^−luc^ cells were obtained from the Center for Translational Medicine, Guangxi Medical University (Nanning, China) and cultured in RPMI 1640 containing 10% fetal bovine serum, 100 U/ml penicillin and 100 μg/ml streptomycin at 37°C and 5% CO_2_.

### Animal

Specific pathogen-free Kunming mice (20 ± 2 g, 4–5 weeks old) and nude male mice (18 ± 2 g, 5–6 weeks old) were purchased from Hunan SJA Laboratory Animal Co., Ltd. (Changsha, China). The mice were maintained in a ventilated room at an ambient temperature of 22 ± 2°C with a 12 h diurnal light cycle. The mice were given ad libitum access to food and water and allowed to acclimatize for 1 week before the experiments. All procedures were approved by the Institutional Animal Ethics Committee of Guangxi Medical University (IAEC, Nos. 201810146, 201903029).

### Extraction and Composition Analysis of Total Flavonoid of Litchi Seed

The Litchi seeds (No. 181001) were purchased from Nanning Shengyuantang Chinese herbal medicine Co., Ltd. (Nanning, China). The voucher specimens (No. 20170713) were identified by Prof. Lilan Qin (Guangxi University of Chinese Medicine) and deposited at the College of Pharmacy of Guangxi Medical University. The seeds were cleaned with distilled water, dried and smashed into powder. Then the TFLS was isolated and purified as described previously (Zhang et al., 2021), and also shown in Supplementary Figure S1. The yield and purity of total flavonoids extract were 3.33 and 42.61% respectively. Then the chemical composition of TFLS was analyzed by ultra-performance liquid chromatography/quadrupole time-of-flight mass spectrometry (UPLC-Q-TOF/MS). The TFLS powder (1 g) was taken in a 10 ml volumetric flask, filled up to the final volume with acetonitrile: water (60:40). The sample concentration was 0.1 g/ml as filtered by a syringe filter (0.22 µm) and stored at 4°C until UPLC-Q-TOF/MS analysis.

Chromatographic analysis was performed on an Acquity UPLC HSS T3 column (100 mm × 2.1 mm, 1.8 μm) by Waters ACQUITY UPLC system (Waters Corp. Milford, MA, United States). The columns were maintained at 30°C and eluted at a flow rate of 0.30 ml/min. The mobile phase was composed of water containing 0.1% formic acid (A) and acetonitrile (B). The gradient program was optimized as follows: 0–1.5 min, 5% B; 1.5–5 min, 5% B to 30% B; 5–13 min, 30% B to 50% B; 13–14 min, 50% B to 5% B, 14–15 min, washing with 5% B. The injection volume was 5 μl.

The mass spectrometry with an electrospray ionization source operating in both positive and negative ion mode was performed on Waters definition accurate mass quadrupole time-of-flight Xevo G2-XS mass spectrometer (Waters Corp., Manchester, United Kingdom). The parameters were set as below: capillary voltage, 3 kV; sample and extraction cone voltage, 40 and 4.0 V; desolvation gas rate and temperature, 600 L/h and 300°C; source temperature, 100 C. Leucine-enkephalin was used as the lock mass in all analyses at a concentration of 400 ng/ml. Data was collected in MSE centroid mode from 50 m/z to 1,500 m/z.

### Cell Proliferation Assay

Cell proliferation was assessed by MTS assay. PC3 and DU145 cells were separately seeded at 3,000 cells/well in 96-well plates and allowed to adhere overnight. Then 100 μl of RPMI-1640 complete medium containing various concentrations (0–240 μg/ml) of TFLS was added to each well and six replicates were used for each treatment concentration. After 24, 48 or 72 h, supernatant was removed and added with100 μl of RPMI-1640 medium. Then, cells were treated with 20 μl of MTS solution and incubated at 37°C for 2 h. Absorbance at 490 nm was determined by a microplate reader and relative cell viability was calculated by comparing treatment group cell viability to the control group. The half-maximal inhibitory concentration (IC_50_) was calculated using SPSS version 20.0.

### Colony Formation Assay

PCa cells (PC3, DU145) were seeded into 6-well plates at a concentration of 400 cells/well and incubated overnight, allowing cell attachment. Then cells were washed twice with phosphate-buffered saline (PBS), RPMI-1640 complete medium containing 50 or 100 μg/ml of TFLS was added to each well. Triplicates were used in each treatment group. Cell status was monitored until multiple macroscopic clones in the culture dish can be observed. Then the cells were fixed with 4% paraformaldehyde and stained with 0.025% crystal violet. After drying, the results of the experiment were imaged with a scanner, then the number of cell clones was calculated by Image J.

### Cell Migration Assay

PC3 and DU145 cells were seeded into 6-well plates and allowed to grow until the cell confluence reaches >95%. Mitomycin at a concentration of 20 μg/ml was then added to the 6-well plate and incubated at 37°C for 2 h. Then a vertical wound was created using a sterile 10 μl pipette tip. Cells were treated with serum-free RPMI-1640 medium containing 50 or 100 μg/ml of TFLS. The same field at each well was imaged with a microscope (ECLIPSE Ti2, Nikon Corporation, Japan) at four time points (0, 8, 16 and 24 h) after treatments. Images were processed and analyzed using Image-Pro Plus software and the scratch areas of each observation point at different times were measured to calculate the cell migration rate.

### Cell Invasion Assay

PC3 and DU145 cells were cultured in the medium containing TFLS for 24 and 48 h before resuspension in a serum-free medium and seeding in the upper transwell chamber. The medium containing 20% FBS was added to the bottom of the lower chamber and allowed for invasion for 24 h. Later, the bottom of the chamber was rinsed with PBS, fixed with 4% paraformaldehyde and stained with 0.025% crystal violet. Then the cells were counted in five random microscopic fields (ECLIPSE Ti2, Nikon Corporation, Japan).

### Flow Cytometry Analysis

Cell sample preparation and staining for Cell cycle and apoptosis analysis by flow cytometry were performed according to the manufacturer’s manual. In brief, PC3 and DU145 cells were cultured in the medium containing 50 or 100 μg/ml TFLS for 24 and 48 h before the cells were harvested. For apoptosis analysis, the PCa cells were washed twice with PBS and resuspended in the Binding Buffer, then Annexin V-FITC and propidium iodide (PI) were added and incubated for 15 min in the dark. Cells were filtered after adding 400 μl 1× Binding Buffer while green fluorescence (Annexin V-FITC) and red fluorescence (PI) were detected by flow cytometry (Accuri C6, BD Biosciences, United States ). In each group, 10,000 cells were analyzed. For cell cycle analysis, the harvested cells were incubated in sequence with solutions A, B, and C. After filtering the cells, the stained cells were analyzed by flow cytometry using FL-2A to score the DNA content of the cells. 10,000 cells were tested for each group.

### TUNEL Assay

PC3 and DU145 cells were harvested after treating with TFLS for 24 and 48 h. Then, the harvested cells were grown on coverslips in a 12-well plate overnight. After washing with PBS, the cells were fixed and permeabilized, followed by TUNEL labeling using a One Step TUNEL Apoptosis Assay Kit. Finally, The DNA-binding dye 4′,6-diamidino-2-phenylindole (DAPI) dihydrochloride was added for nuclear staining. Slides were observed under an inverted fluorescence microscope (ECLIPSE Ti2, Nikon Corporation, Japan). The cells with red fluorescence were defined as apoptotic cells.

### Cell Morphology Assay

PCa cells (PC3, DU145) were inoculated into the cell culture dish and were washed twice with PBS after 24 h. RPMI-1640 complete medium containing 100 μg/ml of TFLS was then added and incubated for 48 h. After TFLS treatment, the morphology of PC3 and DU145 cells was observed using a microscope (ECLIPSE Ti2, Nikon Corporation, Japan). Representative fields were selected for taking pictures.

### Western Blot Analysis

Total cell proteins were extracted and quantified by bicinchoninic acid (BCA) method. Sample proteins were denatured and analyzed by protein electrophoresis using 12% SDS polyacrylamide gel. Then sample proteins were transferred onto polyvinylidene difluoride (PVDF) membranes. After blocking, PVDF membranes were incubated with the corresponding primary antibodies (the dilution ratios of antibodies are showed in Supplementary Table S1) at 4°C overnight before the secondary antibody incubation. With the ECL chemiluminescence kit, the bands were visualized by chemiluminescence/fluorescence/condensation gel imaging analysis system (Champ Chemi 610 Plus, Beijing Saizhi Entrepreneur Technology Co., Ltd., China).

### Immunofluorescence Analysis

After treatment with different concentrations of TFLS, PCa cells (PC3, DU145) were washed twice with PBS and seeded on coverslips in a 12-well plate overnight. After washing with PBS, the cells were fixed in 4% paraformaldehyde for 30 min. Following fixation, the coverslips with PCa cells were washed with PBS three times and then permeabilized with 0.5% Triton X-100 for 15 min at room temperature. Cells were then washed with PBS three more times before incubating with primary antibodies against NF-κB p65 at 4°C overnight. The next day, the slides were stained with corresponding Alexa Fluor^®^ 555-conjugated secondary antibodies for 2 h at room temperature. The cell nuclei were then stained with DAPI for 10 min and examined by fluorescence microscopy (Nikon Intensilight C-HGFI, Nikon Corporation, Japan).

### Animal Model and Treatment

Thirty-two male nude mice were weighed, marked, and allowed for overnight fasting with free access to water. To establish a mouse PCa xenografts model, two million luciferase-expressing PC3 (PC3^−luc^) cells were injected subcutaneously into each mouse’s right flank (day 0). When the primary tumors had reached a mean volume of about 50 mm^3^, the mice were randomly assigned to four groups based on tumor volumes and then treated. According to the clinical application of Lichi seeds in the Chinese Pharmacopoeia and the result of pilot experiment, the dosages of 40 and 80 mg/kg were selected in this experiment. The mice were orally treated with TFLS once daily. Paclitaxel (20 mg/kg), used as a reference drug for positive control, was administered intraperitoneally once per week. The body weight and tumor size of the mice were monitored every 3 days, and *in vivo* bioluminescence imaging was performed once a week by the IVIS imaging systems (Caliper Life Sciences, Hopkinton, MA, United States). At the end of experiment, mice were sacrificed with CO_2_ inhalation. Then tumor volumes and tumor inhibitory rate of each group were respectively calculated using the following formula:

$$\text{Tumor}\;\text{inhibitory}\;\text{rate }=\;\frac{\left(\text{volume}\;\text{in}\;\text{control}\;\text{group}-\text{volume}\;\text{in}\;\text{experimental}\;\text{group}\right)}{\text{volume}\;\text{in}\;\text{control}\;\text{group}}\times 100\text{\%}$$

$$\text{Tumor}\;\text{volumes}\left(\text{V}\right)=1/2\times \left({\text{length×width}}^{2}\right)$$

### Acute Toxicity Test

The acute toxicity test for TFLS was conducted using the maximum-tolerated dose (MTD) method according to Technical Guidelines for Acute Toxicity Test for Traditional Chinese Medicine and Natural Medicine ([Z]GPT2-1, China Food and Drug Administration, 2005). Prior to treatment, animals were weighed, marked, and allowed for overnight fasting with free access to water. Then the mice were randomly assigned to two groups based on their individual body weight. Each group was comprised of five female and five male Kunming mice. 18 g/kg body weight TFLS was orally administered three times on the first day, with an interval of 6 h between doses. The general behavior and body weight of the mice were recorded every 24 h for 14 days. On day 14, all animals were sacrificed with CO_2_ inhalation. Then the vital organs, including the heart, liver, lungs, spleen and kidney, were quickly removed and washed with ice-cold saline. The organs were then weighed, and organ weight index (OWI) was calculated using the following formula:

$$\text{Organ}\;\text{Weight}\;\text{Index}\left(\text{OWI}\right)=\frac{\text{Organ}\;\text{weight}\left(\text{g}\right)}{\text{Body}\;\text{weight}\;\text{of}\;\text{Mice}\;\text{on}\;\text{day}\;\text{of}\;\text{sacrifice}\left(\text{g}\right)}\times 100$$

### Statistical Analysis

Data was presented as mean ± standard error of mean (SEM) of the biological replicates and analyzed by one-way ANOVA followed by LSD or Dunnett’s T3 using SPSS version 20.0. A value of *p* < 0.05 was considered as statistically significant.

## Results

### Analyzing the Components of Total Flavonoid of Litchi Seed

**FIGURE 1:**
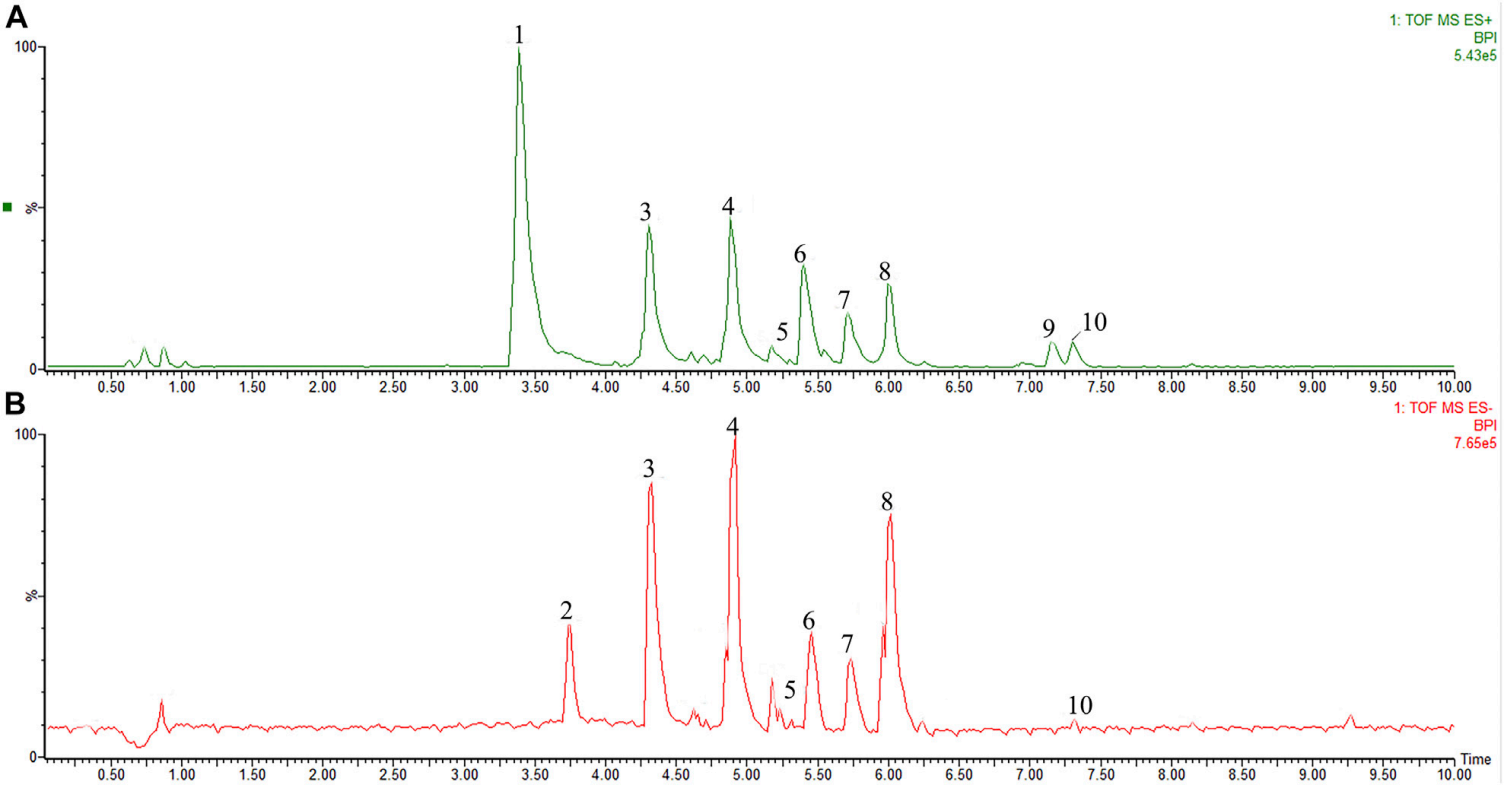
The characterization of TFLS chemical profile. **(A)** positive ion mode; **(B)** negative ion mode; 1) Unknown (C_11_H_18_N_3_O); 2) 2,5-Dihydroxybenzoic acid; 3) 3-O-p-Coumaroylquinic acid; 4) Epicatechin; 5) Rutin; 6) Proanthocyanidin A1; 7) Proanthocyanidin A2; 8) Litchioside. **(C)** 9) Berberine; 10) (-)-Pinocembrin7-O-Neohesperidoside).

The chemical profile of the TFLS was shown in Figure 1. By comparing the m/z values in the databases such as METLIN, Pubchem, Massbank and published literature data, nine components in the TFLS were identified, including 2,5-Dihydroxybenzoic acid, 3-O-p-Coumaroylquinic acid, Epicatechin, Rutin, Proanthocyanidin A1, Proanthocyanidin A2, Litchioside C, Berberine, (-)-Pinocembrin7-O-Neohesperidoside. The detailed information of these components was listed in Table 1 and Supplementary Figure S2.

**TABLE 1:** Characterization and identification of compounds in TFLS.

No.	Name	Molecular formula	Adduction		t_R_/min		*m/z*	
					Positive	Negative	Positive	Negative
1	Unknown	C_11_H_18_N_3_O		—	3.39	—	208.1438→149.0697, 131.0596, 103.0641	—
2	2,5-Dihydroxybenzoic acid	C_7_H_6_O_4_	—	[2M−H]^-^	—	3.733	—	307.0475→153.0208, 109.0298
3	3-O-*p*-Coumaroylquinic acid	C_16_H_18_O_8_	[M+H]^+^	[2M−H]^-^	4.307	4.32	339.1146→321.0978, 293.0988, 147.0546	675.1933→337.0941, 191.0568, 173.0466, 163.0411
4	Epicatechin	C_15_H_14_O_6_	[M+H]^+^	[M−H]^-^	4.88	4.92	291.0964→273.0862, 139.0494, 123.0542	289.0717→245.0764,179.0349,137.0243,125.0253,109.0304
5	Rutin	C_27_H_30_O_16_	[M+H]^+^	[M−H]^-^	5.317	5.300	611.1637→465.1053, 303.0589	609.1454→301.0344
6	Proanthocyanidin A1	C_30_H_24_O_12_	[M+H]^+^	[M−H]^-^	5.440	5.439	577.1346→559.1263, 425.0929, 287.0648	575.1179→557.1047, 423.0716, 271.0624, 137.0239, 125.0239
7	Proanthocyanidin A2	C_30_H_24_O_12_	[M+H]^+^	[M−H]^-^	5.716	5.725	577.1343→425.0920, 287.0649	575.1193→423.0721, 285.0404, 271.0617, 137.0248, 125.0248
8	Litchioside C	C_19_H_34_O_9_	[M+Na]^+^	[M−H]^-^	5.996	6.013	429.2141→407.2322, 245.1842, 241.1525, 227.1741	405.2130→243.1562, 239.1295, 225.1505
9	Berberine	C_20_H_18_NO_4_	[M]^+^	—	7.189	—	336.1302→321.1073, 320.0993, 292.1054	—
10	(-)-Pinocembrin7-O-Neohesperidoside	C_27_H_32_O_13_	[M+H]^+^	[M−H]^-^	7.300	7.317	565.2033→257.0913, 239.0899, 153.0294, 105.0805, 103.0621, 101.0676	563.1774→255.0670

### Total Flavonoid of Litchi Seed Inhibited Proliferation and Viability of Prostate Cancer Cells

**FIGURE 2:**
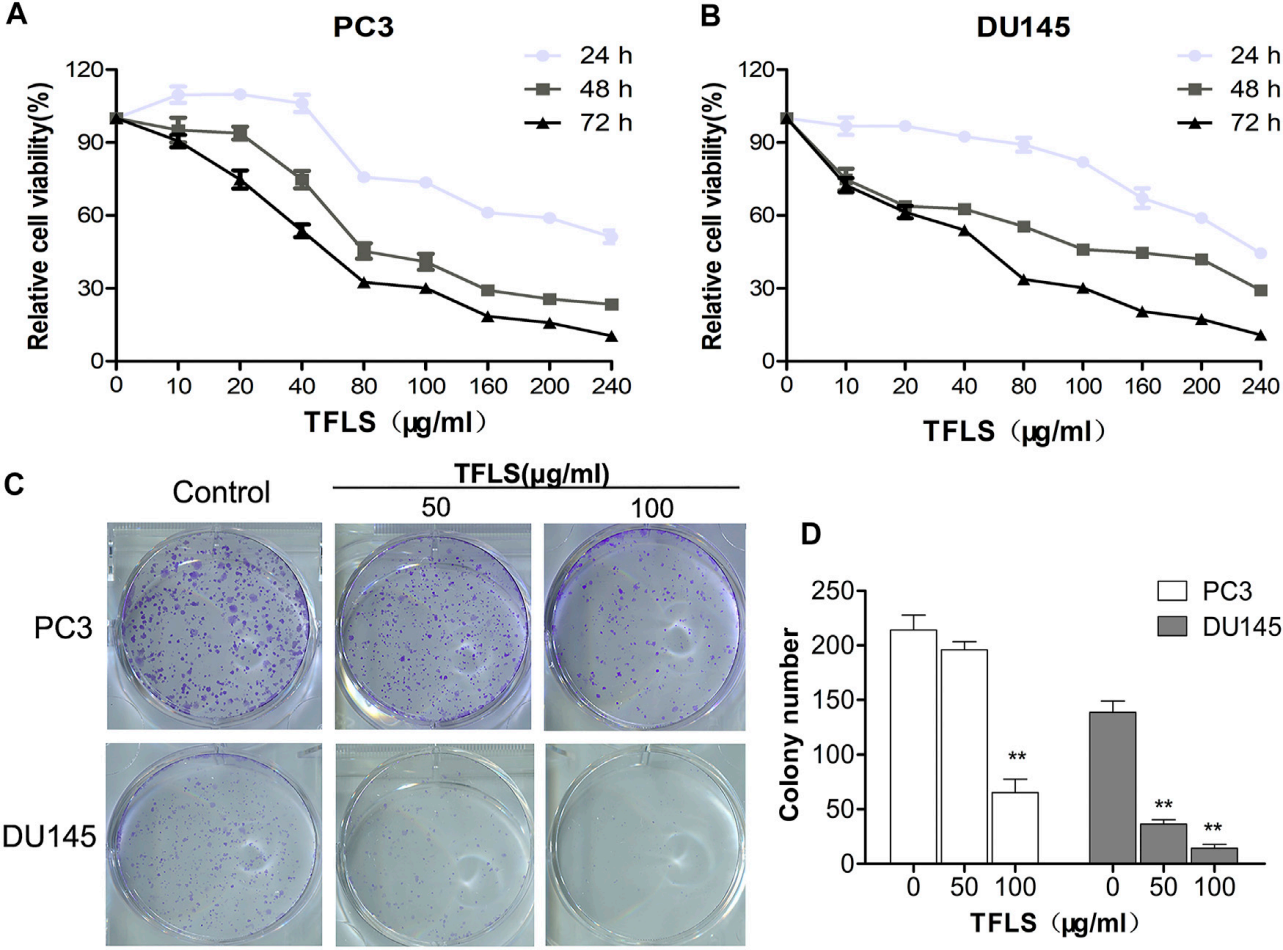
TFLS inhibited the proliferation of PCa cells. **(A,B)** PC3 and DU145 cells were treated with different concentrations of TFLS (0–240 μg/ml) for 24, 48 and 72 h, and cell viability was detected by the MTS assay. **(C)** The colony formation of PC3 and DU145 cells treated with TFLS for 8 days. **(D)** Quantification of the number of colonies of PCa cells. The number of colonies was expressed by mean ± SEM, compared with the control group: *p < 0.05; **p < 0.01.

To investigate the effect of TFLS on cell proliferation, PCa cells were treated with different concentrations of TFLS (0–240 μg/ml) for 24, 48, or 72 h, and the cell viability was evaluated by MTS assay. We found that TFLS significantly inhibited the cell viability of both PC3 and DU145 cells in both dose- and time-dependent manners (Figures 2A,B). The IC_50_ values of TFLS on PC3 and DU145 cells at 72 h were 61.5 ± 7.1 and 55.8 ± 5.1 μg/ml respectively (Table 2).

**TABLE 2:** The IC_50_ value of TFLS on PCa cells.

PCa cells	IC_50_ (μg/ml)		
	24 h	48 h	72 h
PC3	245.2 ± 20.0	95.7 ± 10.6	61.5 ± 7.1
DU145	214.7 ± 15.7	84.6 ± 9.8	55.8 ± 5.1

In addition, we also assessed the effect of various concentrations of TFLS on the proliferative ability of PC3 and DU145 cells by colony formation assay. After 8 days of treatment, the colony-forming ability of DU145 cell was significantly reduced (*p* < 0.01). Compared with the control group, the number of colony formed by PC3 cells was reduced by approximately three quarters after treatment with high concentrations of TFLS (100 μg/ml) (Figures 2C,D).

### Total Flavonoid of Litchi Seed Induced Apoptosis of Prostate Cancer Cells

**FIGURE 3:**
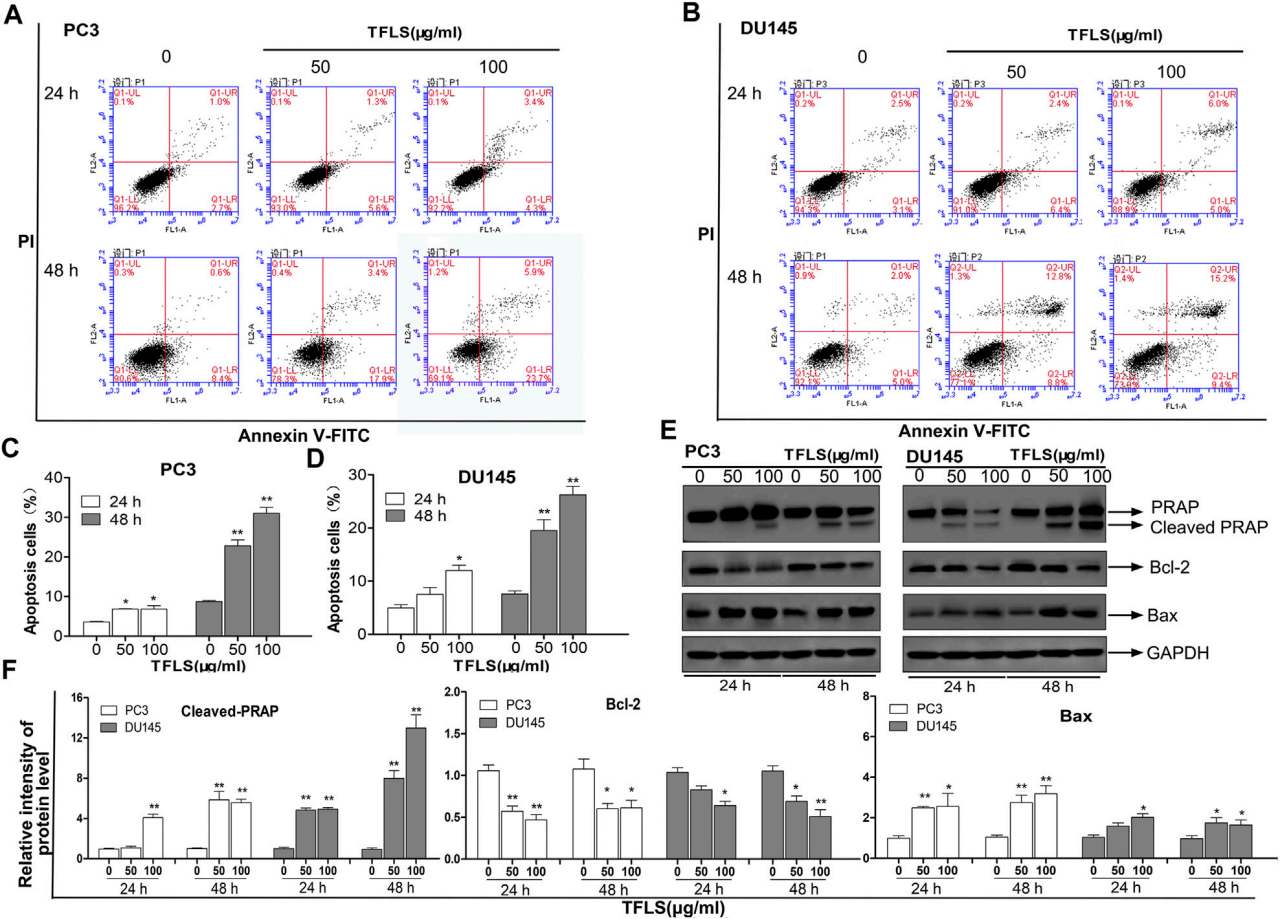
TFLS induced apoptosis in PCa cells. PC3. **(A)** and DU145. **(B)** cells were treated with TFLS, the apoptotic cells were analyzed by Annexin V and PI double staining followed by flow cytometry analysis. The percentage of early apoptotic cells, late apoptotic cells and overall apoptotic cells were determined. Bar plot of overall apoptotic cells in PC3. **(C)** and DU145. **(D)** cells (Annexin V positive cells). **(E)** The expressions of apoptosis-related proteins in PC3 and DU145 cells were evaluated by Western blot. **(F)** Bar plots of the relative expressions of apoptosis-related proteins in PC3 and DU145 cells. Data are expressed as mean ± SEM. Compared with control group: *p < 0.05; **p < 0.01.

**FIGURE 4:**
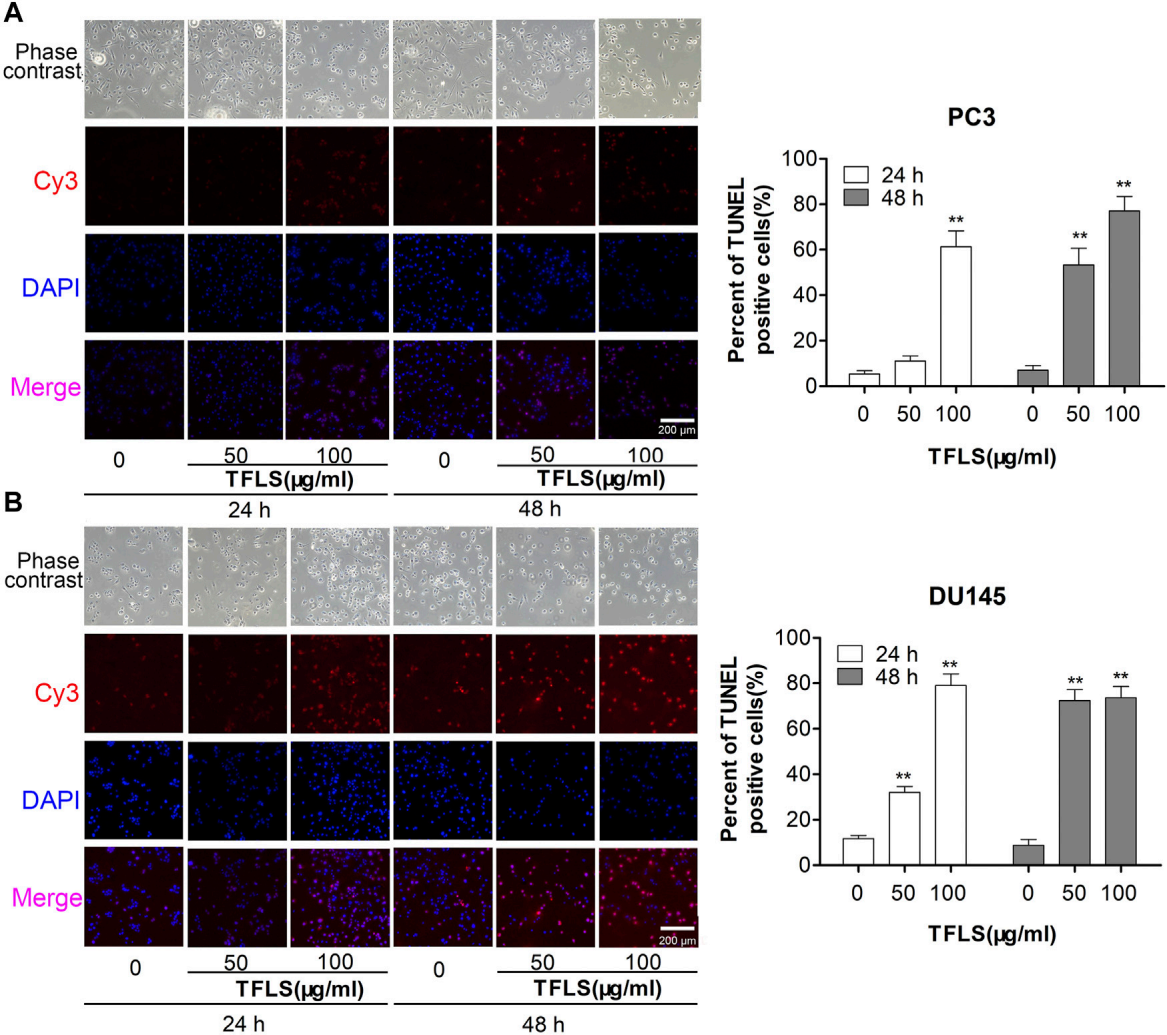
PC3 and DU145 cells were treated with TFLS, and the apoptotic cells were detected by TUNEL assay. Representative images of apoptosis of PC3 and DU145 cells with 0, 50, 100 μg/ml of TFLS treatment for 24 or 48 h. TUNEL positive cells (red) were shown. Cell nuclei were detected by DAPI (blue). The images were obtained by a fluorescence microscope. Bar plots of the percentages of TUNEL positive cells [**(A)** PC3 cells; **(B)** DU145 cells]. Data are expressed as mean ± SEM. Compared with control group: *p < 0.05; **p < 0.01.

To investigate the mechanism by which TFLS inhibited the PCa cell proliferation, we first assessed the effect of TFLS on cell cycle and apoptosis using flow cytometry analysis. The results (Figures 3A–D) showed that the percentage of apoptotic PC3 and DU145 cells was significantly higher in the TFLS treatment groups than that of the control groups. After 48 h treatment, the apopttic rates of PC3 and DU145 cells treated with 100 μg/ml TFLS were 31.10 ± 1.17% and 26.23 ± 1.34%, respectively. These results illustrated that TFLS might promote apoptosis of PCa cells. We further confirmed the effect of TFLS on cell apoptosis by TUNEL assay. We found that the number of TUNEL-positive cells was significantly increased in both the PC3 and DU145 cells treated with TFLS compared to the un-treated cells (Figure 4). These results suggested that TFLS could induce apoptosis of PCa cells.

To explore the mechanisms underlying TFLS-induced apoptosis of PCa cells, we screened the expression profiles of a panel of proteins involved in cellular apoptosis using Western blot. The data showed that TFLS increased the expression of cleaved- Poly ADP-ribose polymerase (PARP) and Bcl-2-associated X protein (Bax), while decreased the expression of B-cell lymphoma-2 (Bcl-2) in both PC3 and DU145 cells (Figures 3E,F). We also detected the effect of TFLS on cell cycle, and found that the cell cycle was not affected by TFLS treatment in these 2 cell lines (Supplementary Figure S3).

### Total Flavonoid of Litchi Seed Inhibited Prostate Cancer Cell Migration and Invasion

**FIGURE 5:**
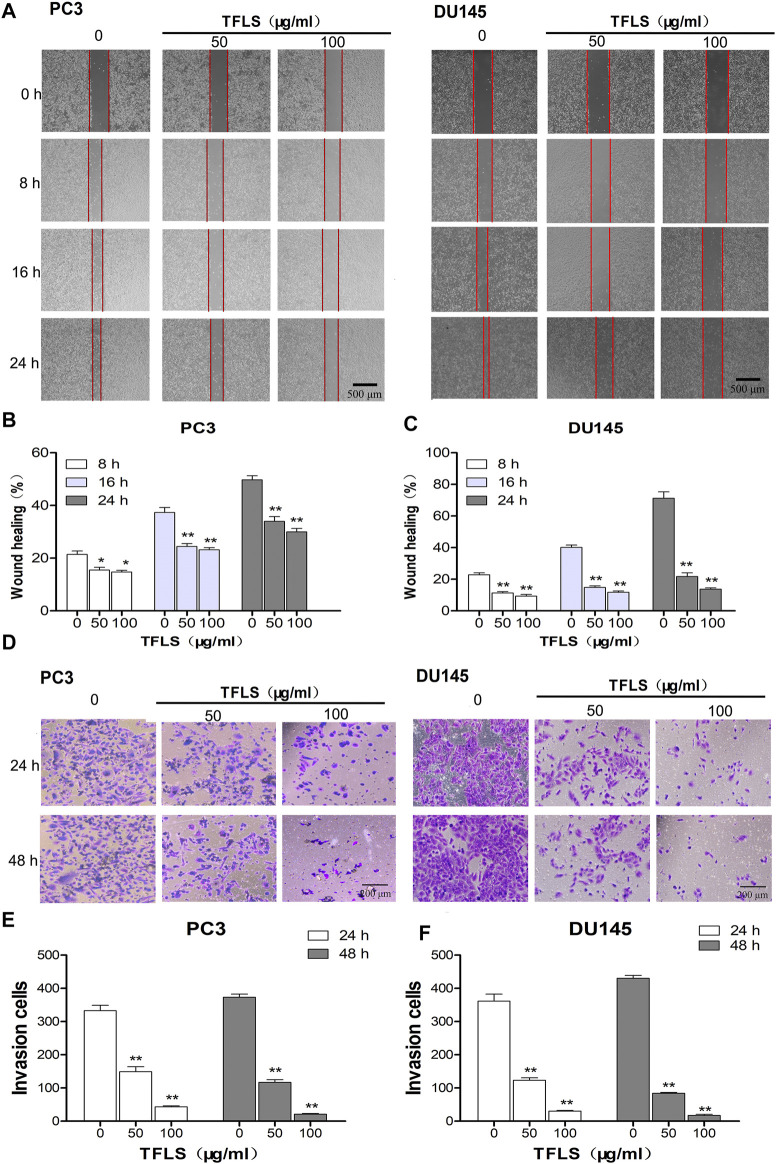
TFLS inhibited the metastasis of PCa cells. **(A)** The effects of TFLS on the migration of PC3 and DU145 cells were analyzed using wound-healing assay. **(B,C)** Quantitative analysis of average wound healing degrees of PC3 and DU145 cells. **(D)** Invasiveness of PC3 and DU145 cells that underwent TFLS treatment determined by transwell invasion assay. **(E,F)** Invaded PC3 and DU145 cells were quantified; Data are expressed as mean ± SEM, compared with the control group: *p < 0.05; **p < 0.01.

In order to examine the effect of TFLS on metastasis in PCa cells, we firstly tested the cell migratory ability of PC3 and DU145 cells by wound-healing assay. As shown in Figures 5A–C, TFLS significantly reduced the gap closure rate. The percentage of PC3 cell migration reached 50% in the control group, the migratory rate was 29.4 ± 1.3% in cells treated with 100 μg/ml TFLS. Interestingly, TFLS showed a more potent inhibitory effect on DU145 cells after 24 h treatment. The migratory rate was reduced to 21.6 ± 2.3% in DU145 cells treated with 50 μg/ml of TFLS, which was further decreased to 13.6 ± 0.8% at 100 μg/ml. Similarly, it was found that TFLS reduced cell invasion in a dose-dependent manner in the transwell assay (Figures 5D–F).

### Total Flavonoid of Litchi Seed Inhibited Epithelial-to-Mesenchymal Transition in Prostate Cancer Cells

**FIGURE 6:**
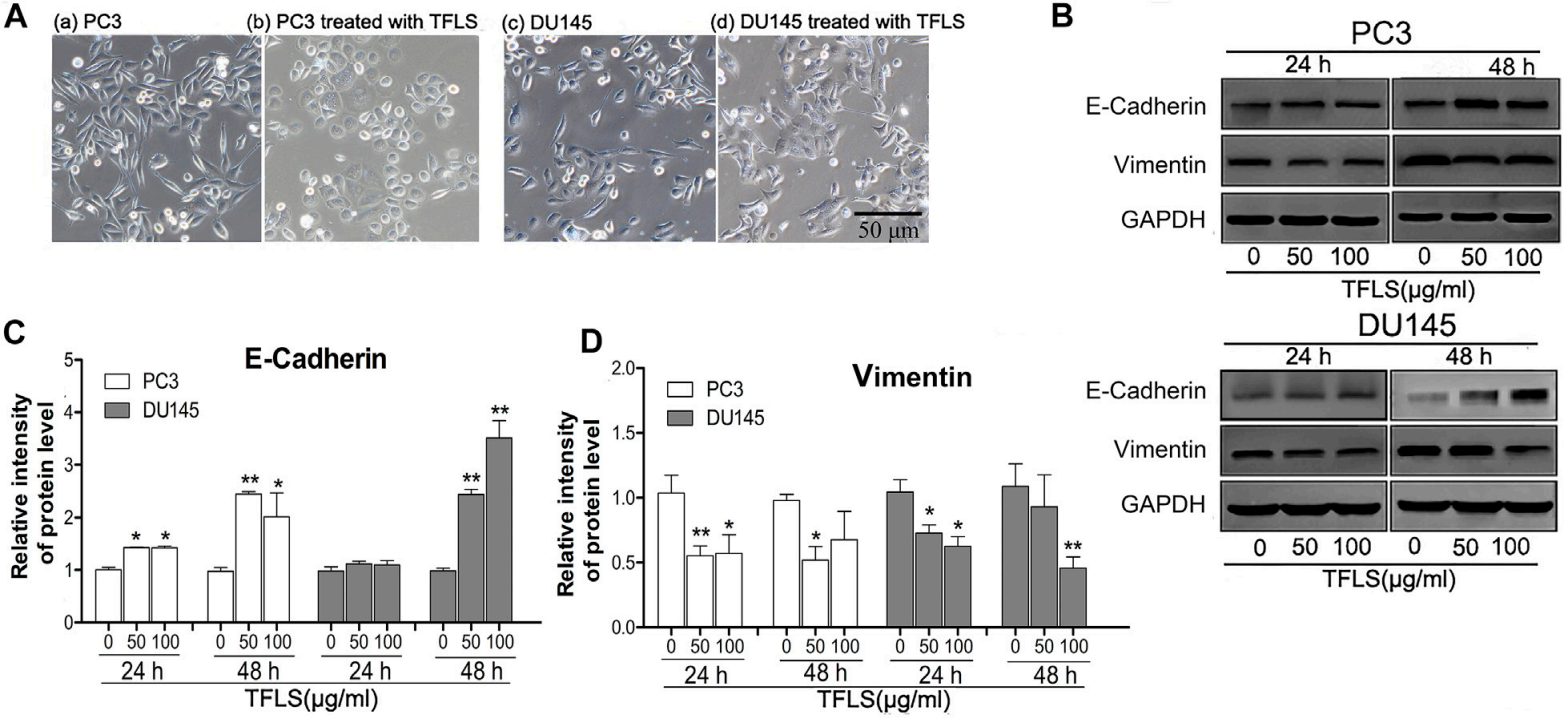
TFLS inhibited EMT in PCa cells. **(A)** The morphology of PC3 and DU145 cells treated with or without TFLS (100 μg/ml) for 48 h (magnification 400×). **(B)** The expression of EMT-related proteins in PC3 and DU145 cells with 0, 50, 100 μg/ml of TFLS treatment for 24 or 48 h; Relative expression of E-cadherin **(C)** and Vimentin **(D)** was evaluated by ImageJ and shown as bar plots. Data are expressed as mean ± SEM, compared with the control group: *p < 0.05; **p < 0.01.

EMT is one of the hallmarks of cancer metastasis. We investigated the inhibitory effects of TFLS on the EMT. We first observed the effect of TFLS on cell morphology. It was found that the un-treated PC3 and DU145 cells displayed a mesenchymal-like elongated spindle shape with sparse distribution, with observation of pseudopodium on some cells. However, after treating with TFLS, cells adapted to a more rounded shape accompanied by disappearance of pseudopodium (Figure 6A). These results suggested that TFLS might inhibit EMT in PCa.

To confirm the effect of TFLS on EMT, we evaluated the expression of EMT biomarkers, including E-cadherin and Vimentin. It was found that the expression of epithelial biomarker E-cadherin increased, while mesenchymal biomarker Vimentin’s expression reduced after TFLS treatment compared with the control group, (Figures 6B–D). These results indicated that TFLS reduced migratory and invasive capabilities in PC3 and DU145 cells via phenotypic inversion of EMT.

### Total Flavonoid of Litchi Seed Regulated AKT/mTOR and NF-κB Signaling Pathways

**FIGURE 7:**
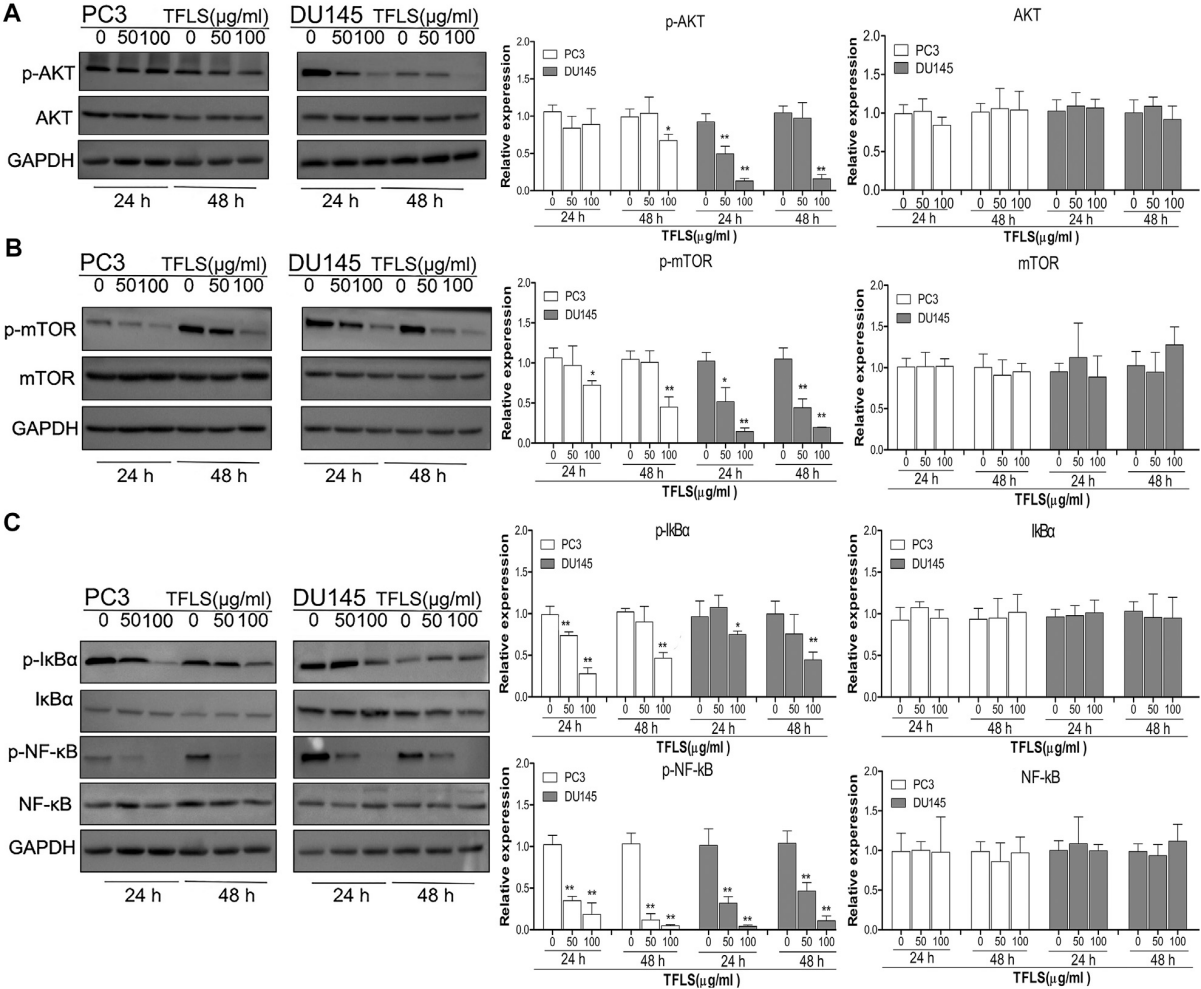
TFLS inhibited the AKT/mTOR and NF-κB signaling pathways in PCa cells. PC3 and DU145 cells were treated with TFLS, the expression of key proteins in AKT pathway **(A)**, mTOR **(B)**, NF-κB **(C)** signaling pathways was analyzed by Western blot. GAPDH was used as a control. Data are expressed as mean ± SEM, compared with the control group: *p < 0.05; **p < 0.01.

**FIGURE 8:**
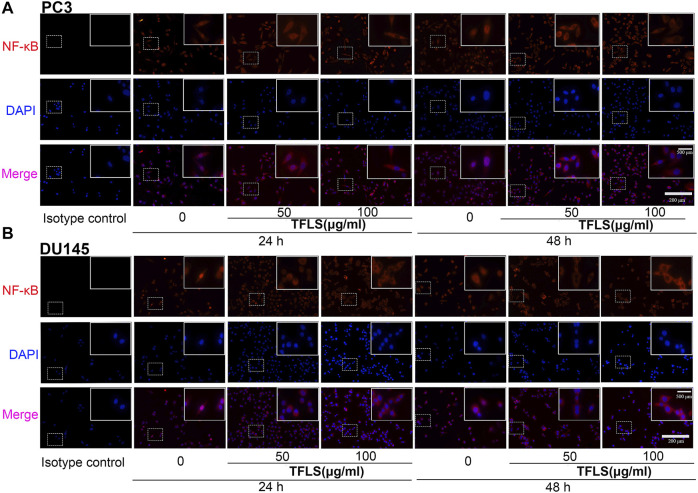
TFLS inhibited the nucleus expression of NF-κB in PCa cells. **(A–B)** Representative images of immunofluorescence staining showing NF-κB p65 (red) in PC3 and DU145 cells treated without or with TFLS (50, 100 μg/ml). Cell nuclei were detected by DAPI (blue). The images were obtained by fluorescence microscope.

To further explore the underlying mechanisms of TFLS, we examined the expression of key proteins in signaling pathways implicated in cancer cell viability, proliferation and metastasis. For example, AKT, AMPK, MAPK and Wnt signaling pathways. We found that TFLS has little impact on p-AMPKɑ, Wnt3a, p-MEK1/2 and p-P44/42 MAPK (Supplementary Figure S4). Interestingly, it was evidenced that the phosphorylation of AKT was effectively inhibited by TFLS in both PC3 and DU145 cells (Figure 7A), which may lead to changes in expression of several downstream proteins in the AKT signaling pathway. Hence, the total levels and activities of proteins involved in the AKT pathway were explored by Western blot. As shown in Figures 7B,C, after TFLS treatment, the levels of p-IκB α, p-NF-κB and p-mTOR in both PC3 and DU145 cells were significantly reduced compared to the control group. Notably, TFLS had no effect on the total expressions of AKT, IκB α, NF-κB and mTOR. The inhibitory effect of TFLS on the activation of NF-κB was further confirmed by immunofluorescence study. As shown in Figure 8, NF-κB p65 was mostly localized in the nucleus in the control group, as compared to a dispersed expression pattern in the treatment group.

### Total Flavonoid of Litchi Seed Suppressed Tumor Growth of PC3 Xenografts *In Vivo*

**FIGURE 9:**
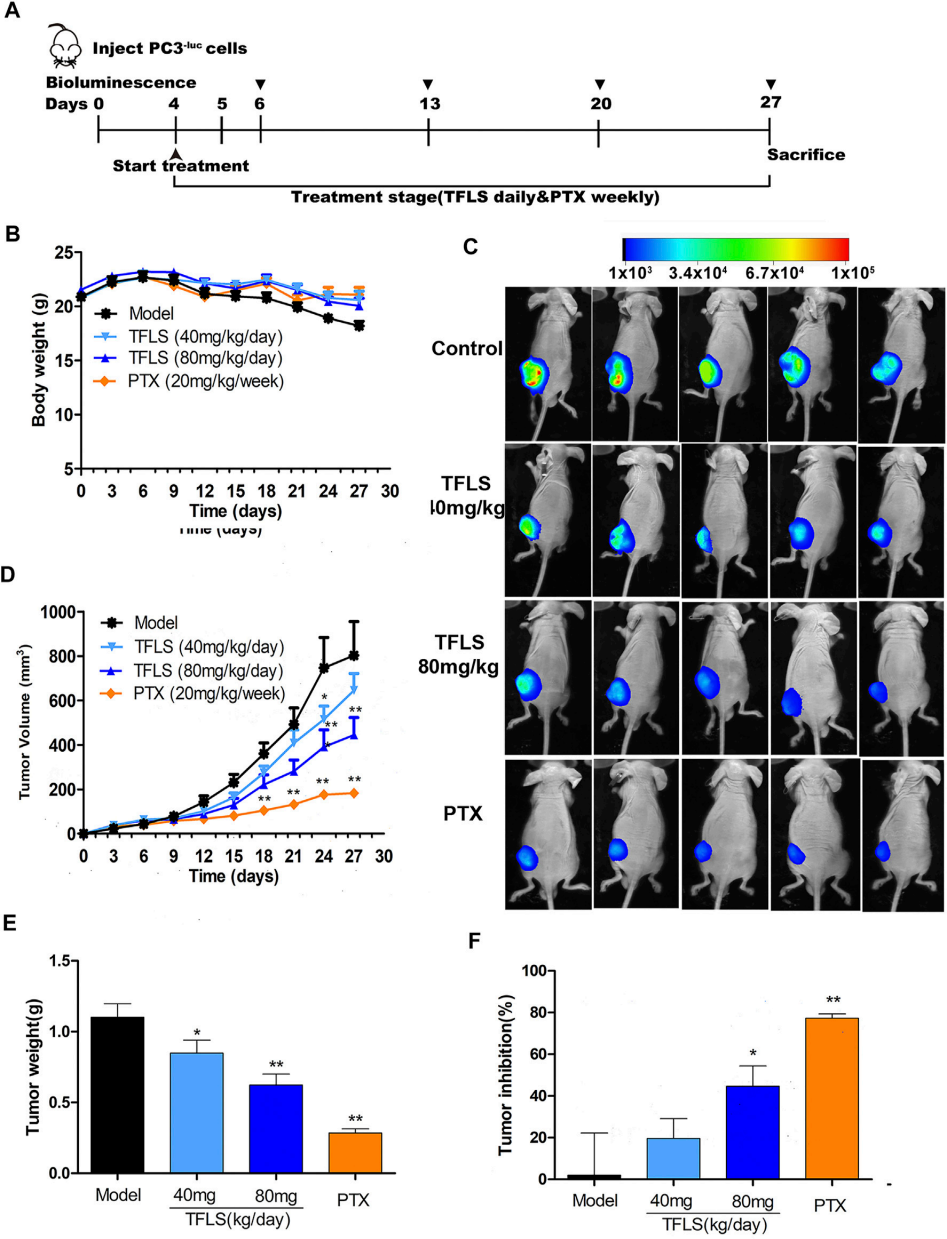
TFLS suppressed tumor growth of PC3^−luc^ xenografts *in vivo*. **(A)** Outline of the animal experiment. **(B)** Body weight curves of mice in different groups during the whole treatment course. **(C)** Representative bioluminescence imaging of PC3^−luc^ xenograft tumors in different groups at the end of experiments. **(D)** Tumor growth curves of mice in different groups during the whole treatment course. **(E)** Tumor weight in different groups at the end of experiments. **(F)** Tumor inhibition rates in different groups at the end of experiments. Data are presented as mean ± SEM. Compared to control group: *p < 0.05. **p < 0.01. PTX: Paclitaxel.

To determine whether TFLS inhibits tumor progression *in vivo*, we established a nude mouse PC3^−luc^ xenografts model. After treatment with TFLS, there was no significant difference in body weight between the control group and TFLS treatment group (Figure 9B). However, the tumor weight and volume of TFLS-treated mice (at the dose of 40 and 80 mg/kg) was significantly lower than untreated mice (Figures 9D,E). Administration of either 40 or 80 mg/kg body weight TFLS showed a significant antitumor activity with tumor inhibition rates of 20.87 and 44.63%, respectively (Figure 9F). The bioluminescence imaging of PC3^−luc^ xenograft tumors in different groups at the end of experiments showed consistent results (Figure 9C).

### Acute Toxicity Test

**FIGURE 10:**
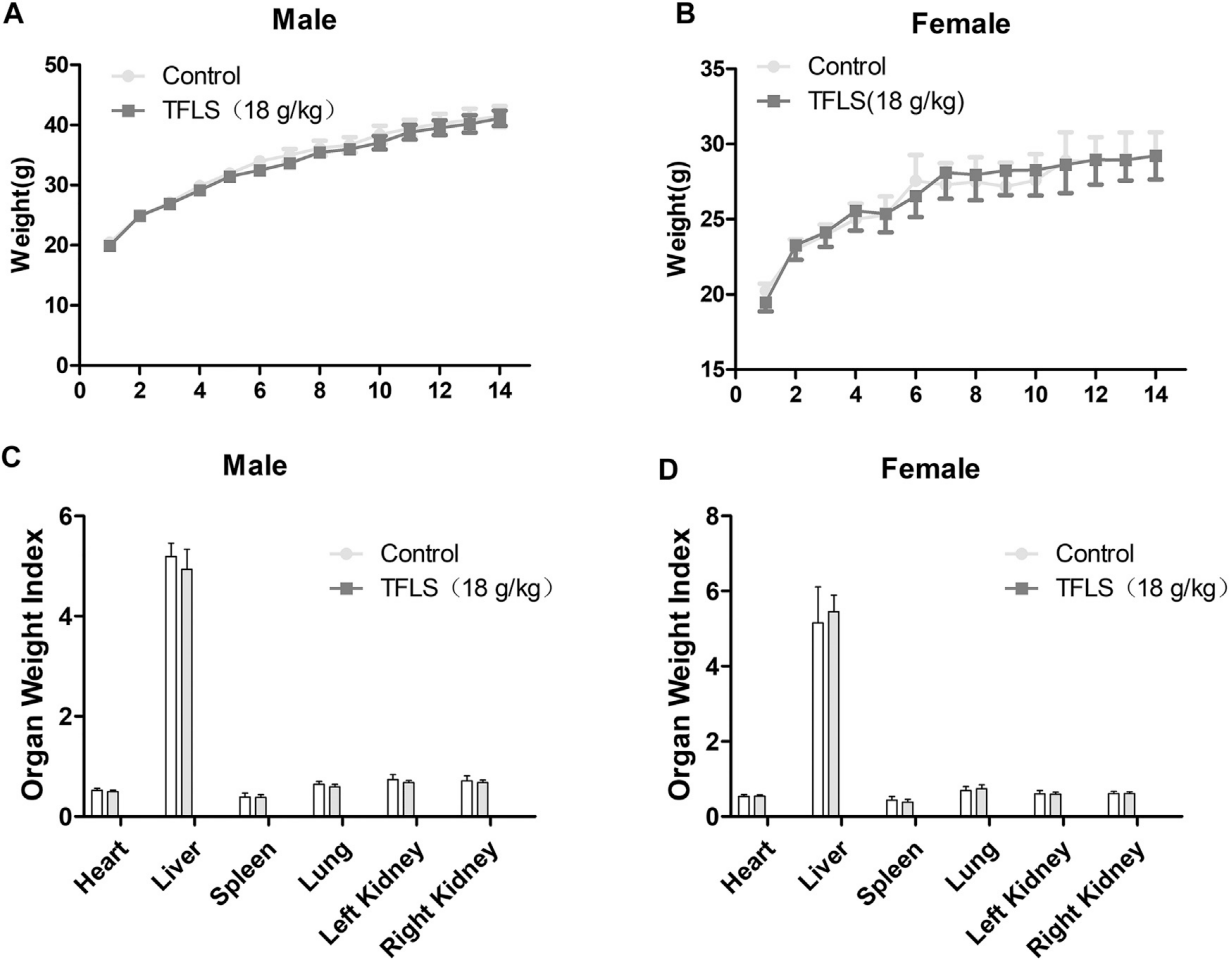
The effects of TFLS on body weight and OWI. TFLS (18 g/kg body weight) was orally administered three times on the first day, with intervals of 6 h. The body weight was measured every day for 14 days in the male **(A)** and female **(B)** mice. On day 14, all animals were sacrificed. The vital organs were weighed and OWI was calculated **(C,D)**. Data are expressed as mean ± SEM.

Fourteen days after oral TFLS, no toxicity-related clinical symptom or mortality were observed. As shown in Figures 10A,B, there was no significant change in body weight by TFLS treatment in both male and female mice. In addition, no significant pathological change was observed in the textures, colors and organ weight index of vital organs, including the heart, liver, spleen, lung and kidney (Figures 10C,D). These results demonstrated that the median lethal dose (LD_50_) of TFLS in mice is well-above 18 g/kg.

## Discussion

PCa is the second leading cause of cancer-related male mortality in Western countries. PCa is difficult to be treated with current clinical treatment modalities (Siegel et al., 2019). Hence, there is an urgent demand for novel anti-PCa therapies. Litchi seeds have been traditionally used in Chinese herbal formulas for urologic neoplasms including prostate cancer, renal carcinoma and bladder cancer (Hsu et al., 2012; Emanuele et al., 2018). In a previous study, we found that flavonoids were the most abundant chemical component among the extracts of Litchi seeds which exerted anti-PCa activity (Guo et al., 2017; Cao et al., 2020). Other studies also found that flavonoids extracted from Litchi pericarp such as epicatechin and proanthocyanidin B2 had anticancer activities (Koga et al., 2017). Therefore, in this study, we investigated the anticancer effects of TFLS on PCa cells. The results showed that TFLS significantly inhibited the proliferation and colony formation capability of PCa cells *in vitro*. More importantly, we also confirmed that TFLS could suppress tumor growth using a nude mouse PC3^−luc^ xenograft model *in vivo*. Meanwhile, in the acute toxicity test, neither mortality nor abnormalities were detected in mice that were orally administered with 18 g/kg TFLS. The LD_50_ of TFLS in mice is well-above 18 g/kg. These findings demonstrated that TFLS might be a potent anti-tumor therapeutics with low toxicity and limited side effects. We investigated the molecular mechanisms of anti-PCa activity of TFLS *in vitro*. It was found that TFLS displays a dose- and time-dependent inhibitory effect on human breast cancer, which could be attributed, in part, to its regulation of apoptosis in cancer cells through upregulation and downregulation of multiple genes (Li and Jiang, 2007). So, we examined the effect of TFLS on cell cycle and apoptosis by flow cytometry and found that TFLS could induce apoptosis of PCa cells, which was further verified by TUNEL assay. In addition, our results showed that TFLS had little regulatory effect on the cell cycle. These findings indicated that the anti-proliferative function of TFLS on PCa cells was likely to be majorly exerted by apoptosis induction. So, we focused on the effect of TFLS on apoptosis-related proteins. The Bcl-2 family proteins are major regulators of the intrinsic apoptosis pathway. Among them, Bax was identified as a homologous binding partner of Bcl-2 that activates apoptosis (Boise et al., 1995). In contrast, PARP maintains cell viability (Satoh and Lindahl, 1992); Cleaved-PARP promotes cell disintegration and is considered as a biomarker of apoptosis (Oliver et al., 1998). Interestingly, in our study, we confirmed that TFLS treatment increased the expression of Bax and Cleaved-PRAP, while decreased the expression of Bcl-2, indicating that TFLS may induce intrinsic apoptosis by regulating the expression of Bcl-2 and Bax.

Tumor metastasis is one of the major characteristics of malignant tumors and remained an obstacle in cancer treatment. EMT is an essential process underlying metastasis, during which epithelial cells gradually gain mesenchymal properties with enhanced migratory and invasive capabilities (Huber et al., 2005). Because previous studies have shown EMT was an essential process underlying PCa metastasis, we examined if TFLS could prevent metastasis by inhibiting EMT. The changes in cellular properties and a group of molecular biomarkers are the main criteria for defining EMT status (Ievgenia and Cédric, 2019). In this study, we found that PC3 and DU145 cells gained epithelial property when treated with TFLS, suggesting that TFLS could prevent changes of cell morphology during EMT. We tested the expression of EMT protein biomarkers, including E-cadherin and Vimentin. E-cadherin is majorly expressed in epithelial cells to maintain cell-cell contact (Hay and Zuk, 1995), the downregulation of which was observed in embryogenesis, tissue fibrosis and cancer-related EMT. While the upregulation of Vimentin is strongly associated with tumor cell invasion and metastasis (Raymond and Leong, 1989). We found that TFLS significantly upregulated the expression of epithelial biomarker E-cadherin and inhibited the mesenchymal biomarker Vimentin. We confirmed that TFLS has an inhibitory effect on metastasis of PCa cells by preventing EMT based on the changes in cellular properties and expression of molecular biomarkers.

Furthermore, we tested the activation of signaling pathways that are highly associated with cell proliferation and metastasis upon TFLS treatment, including AKT, AMPK, MAPK and Wnt signaling pathways. AKT signaling pathway is critically implicated in both pathogenesis and development of PCa, the inhibition of which could induce cell apoptosis and inhibit metastasis (Tang et al., 2013). Upon stimulation and subsequent phosphorylation, AKT translocates into the nucleus and regulates target gene expression which is related to cancer cell viability, proliferation, apoptosis, cell cycle progression, metastasis and vasculogenesis (Chen H. et al., 2016). We found that TFLS could significantly decrease the phosphorylation of AKT in PC3 and DU145 cells. On the contrary, other signal pathways like AMPKɑ, MEK1/2, P44/42 MAPK and Wnt 3a were not found to be affected by TFLS, suggesting that the anti-PCa property of TFLS was majorly exerted by inhibiting AKT signaling pathway. Hence, we hypothesized that TFLS induced apoptosis and inhibited metastasis of PCa cells by inhibiting the AKT signaling pathway.

**FIGURE 11:**
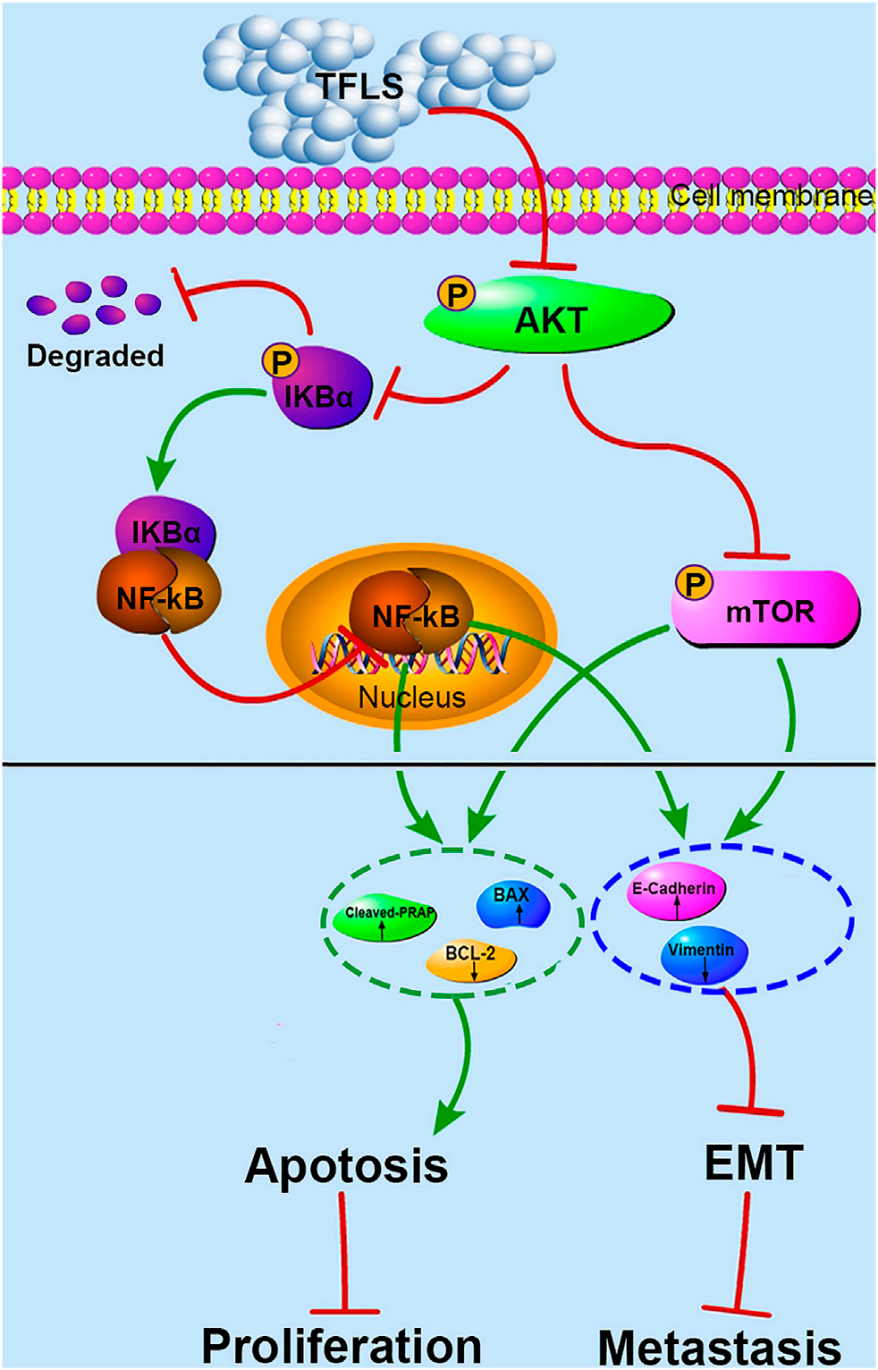
Proposed mechanism of TFLS anti-PCa cells. In brief, TFLS inhibited the phosphorylation of AKT after entering cells, which resulted in decreased phosphorylation of mTOR and IκBα. Then, IκBα and NF-κB combined to form complex of NF-κB-IκBα, which suppressed NF-κB to translocate into the nucleus. The reduction of mTOR phosphorylation and inhibition of NF-κB’s nuclear translocation thus regulated the expression of apoptosis- and EMT-related proteins. As a result, cancer progression was suppressed.

To investigate the direct effect of TFLS on AKT, we tested the expression of the downstream proteins in this signaling pathway to further confirm the mechanism. IKK proteins, including IKK-α and IKK-β, could be activated by phosphorylated AKT. Their activation induces the phosphorylation and subsequent degradation of IκBα, leading to the dissociation of IκBα/NF-κB complex, nuclear translocation and activation of NF-κB signaling pathway (Chandrasekar et al., 2004). In our study, we found that TFLS inhibited NF-κB signaling pathway by decreasing the phosphorylation of IκBα and nucleus expression of NF-κB. This is supported by similar results showing that TFLS could attenuate the nuclear translocation of NF-κB in activated HSC-T6 (Qin et al., 2018). Meanwhile, our study confirmed that TFLS reduced the expression of phosphorylated NF-κB, which may also be resulted from the downregulation of NF-κB signaling pathway. NF-κB signaling pathway is highly associated with EMT and can promote a mesenchymal cell phenotype (Min et al., 2008). The activation of NF-κB promotes its binding to the VIM promoter, which upregulates Vimentin expression thus adaptation to a more mesenchymal phenotype (Lilienbaum and Paulin, 1993). Meanwhile, activated NF-κB inhibited the expression of E-cadherin by elevating transcriptional repressors of E-cadherin in multiple cancer types (Chua et al., 2007). Our study illustrated that NF-κB influenced EMT by regulating EMT-related proteins. In addition, it is well-established that the suppression of NF-κB could also promote cell apoptosis by upregulating pro-apoptosis proteins while downregulating anti-apoptosis proteins. Bcl-2 is transcriptionally regulated by NF-κB and this activity is mediated through a functional NF-κB site identified in the Bcl-2 p2 promoter in PCa cell lines (Catz and Johnson, 2001). NF-κB also regulates Bax gene expression through an indirect pathway by inducing PRAP caspase-3-mediated cleavage (Bentires-Alj et al., 2001). In accordance with the above findings, our study showed that TFLS increased the level of cleaved-PRAP and Bax, and reduced the level of Bcl-2. Hence, our results demonstrated that TFLS exerted pro-apoptosis and anti-metastatic effects in PCa cells by inhibiting the activation of AKT/NF-κB signaling pathway (Figure 11).

The mTOR is another important target protein downstream of the AKT signaling pathway and phosphorylated ATK could directly activate mTOR (Spirina et al., 2018). Interestingly, in this study, we also found that TFLS decreased phosphorylation level of mTOR. This finding implied that TFLS could inhibit AKT/mTOR signaling pathway. It has been reported that the mTOR signaling pathway plays a key role in the migration, invasion and EMT of PCa. Vo et al. (2013) confirmed that overactivation of AKT/mTOR signaling pathway enhanced the migration capability of PCa cells. We found that TFLS negatively regulates EMT, which could be mediated by inhibition of AKT/mTOR signalling pathway. It is reported that inhibition of Akt/mTOR reversed EMT by regulating the EMT-related proteins (Karimi Roshan et al., 2019). Our study also implied that the expression of E-Cadherin and Vimentin were regulated by AKT/mTOR pathway after TFLS treatment. Notably, activation of mTOR signaling pathway is also highly associated with tumor cell apoptosis, as evidenced by (Su et al., 2019). AKT/mTOR signaling pathway regulates the expression of apoptosis-related proteins, including Bcl-2, Bax and PRAP, etc (Xia et al., 2020). Therefore, we speculated that TFLS induced the apoptosis of PCa cells by inhibiting AKT/mTOR signaling pathway (Figure 11).

## Conclusion

In summary, this study provides new insights into the role of TFLS as an anti-cancer agent in PCa. We show that TFLS could suppress the PCa growth *in vivo* and inhibit PCa cells proliferation, migration and invasion *in vitro* through induction of apoptosis and phenotypic inversion of EMT, which may be realized by inhibiting AKT/mTOR and NF-κB signaling pathways.

## Data Availability

The original contributions presented in the study are included in the article/Supplementary Material, further inquiries can be directed to the corresponding authors.
