# Supplementary material for: Total Flavonoids of Litchi Seed Attenuate Prostate Cancer Progression Via Inhibiting AKT/mTOR and NF-kB Signaling Pathways
Source: Front Pharmacol. 2021 Sep 23;12:758219. doi: 10.3389/fphar.2021.758219 (PMC8495171; doi:10.3389/fphar.2021.758219)
Supplement: Supplementary file 1 [file Presentation1.pdf]

Supplementary Material

1 Supplementary Figures and Tables

1.1 Supplementary Figures

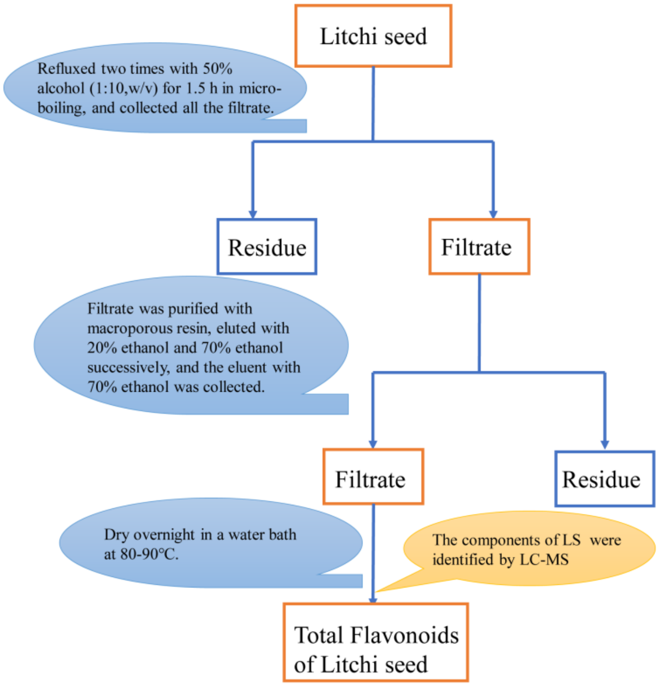

Supplementary Figure 1. The flow chart of the extraction process of TFLS. The Lichi seeds were dried and smashed into powder, then refluxed with 50% ethanol at 1:10 (w/v) for 1.5 h, repeated the procedure twice, the filtrate was collected and concentrated with rotary evaporator. Then, the concentrated filtrate was purified with macroporous resin (HPD-BJQH), eluted with 20% ethanol for 1h and then with 70% ethanol for 1h. The eluent with 70% ethanol was collected and dried overnight at 80-90 °C. The extracts were stored in freezer under -20 °C before used.

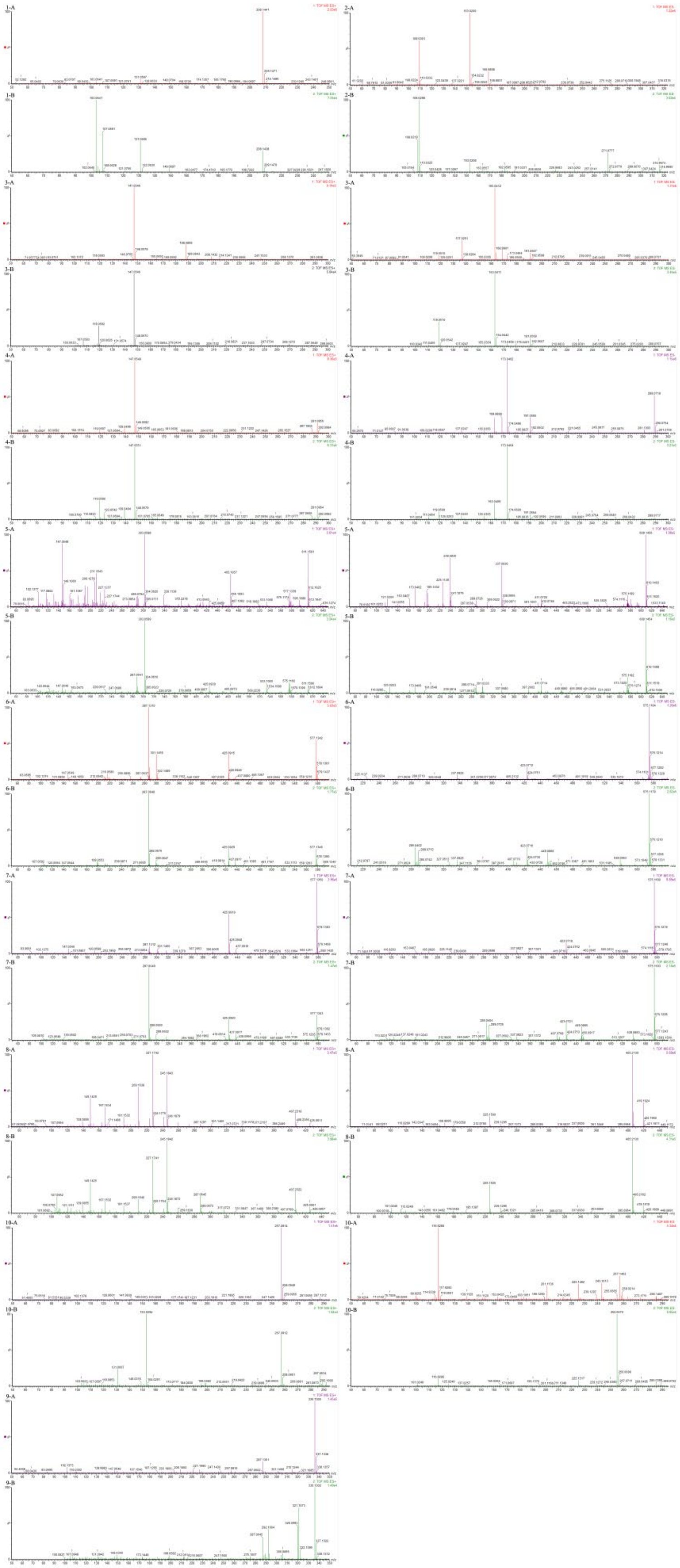

Supplementary Figure 2. The characteristic centroid mass spectrum of TFLS chemical profile. (Left: positive ion mode, Right: negative ion mode; A: MS centroid; B: MS2 centroid; 1: Unknown ($C_{11}H_{18}N_3O$); 2: 2,5-Dihydroxybenzoic acid; 3: 3-O-p-Coumaroylquinic acid; 4: Epicatechin; 5: Rutin; 6: Proanthocyanidin A1; 7: Proanthocyanidin A2; 8: Litchioside C; 9: Berberine; 10: (-)-Pinocembrin7-O-Neohesperidoside;)

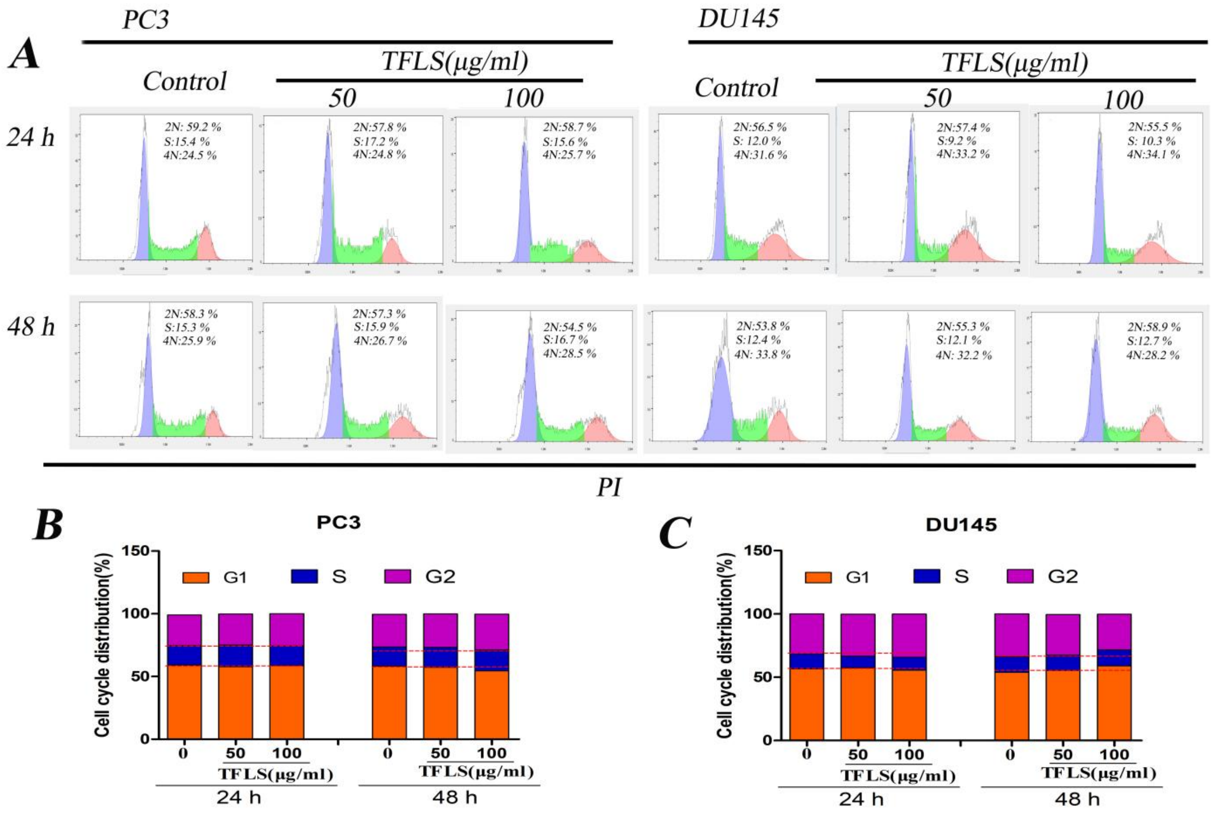

Supplementary Figure 3. The effect of TFLS on cell cycle of PCa cells. (A) PC3 and DU145 cells were treated with TFLS for 24 and 48h, and cell cycle was detected by Flow cytometry; (B) Bar plot of the cell cycle distribution of PC3; (C) Bar plot of the cell cycle distribution of DU145.

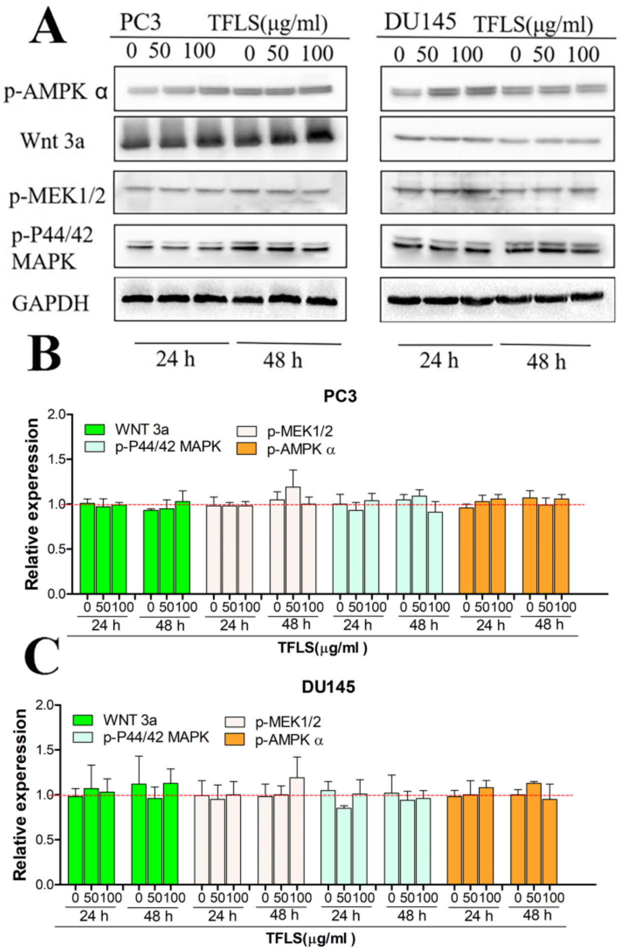

Supplementary Figure 4. The effects of TFLS on the AMPK, Wnt and MAPK signaling pathways in PCa cells. (A) PC3 and DU145 cells were treated with TFLS, the levels of p-AMPK α, Wnt 3a, p-MEK1/2 and p-P44/42 MAPK were analyzed by western blot; GAPDH was used as a loading control. (B-C) Bar plot of the relative expression of Wnt 3a, p-MEK1/2, p-P44/42 MAPK and p-AMPK α in both PC3 and DU145 cells. Data are presented as mean ± SEM.

1.2 Supplementary Table

Table 1

Antibodies used in western blot analysis

Antibodies	Item.No.	Dilution ratio	Company
AKT	4691	1:1000	CST
E-cadherin	3195	1:1000	CST
GAPDH	97166	1:2000	CST
IκB α	9242	1:1000	CST
mTOR	2983	1:1000	CST
NF-κB p65	8242	1:1000	CST
p-AKT(Ser473)	4060	1:1000	CST
p-AMPKα(Thr172)	50081	1:1000	CST
p-IκBα (Ser32)	2859	1:1000	CST
p-MEK1/2(Thr286)	9127	1:1000	CST
p-mTOR(Ser2448)	5536	1:1000	CST
p-NF-κB p65(Ser468)	3039	1:1000	CST
p-p44/42 MAPK(Thr202/Tyr204)	4370	1:1000	CST
Vimentin	3390	1:1000	CST
Wnt 3a	ab28472	1:2000	Abcam
β-Catenin	8480	1:1000	CST
