# Supplementary material for: Total Flavonoids of Litchi Seed Attenuate Prostate Cancer Progression Via Inhibiting AKT/mTOR and NF-kB Signaling Pathways
Source: Front Pharmacol. 2021 Sep 23;12:758219. doi: 10.3389/fphar.2021.758219 (PMC8495171; doi:10.3389/fphar.2021.758219)

Original of Western Blot

(The original document is processed by cutting and matching colors)

Apoptosis marker

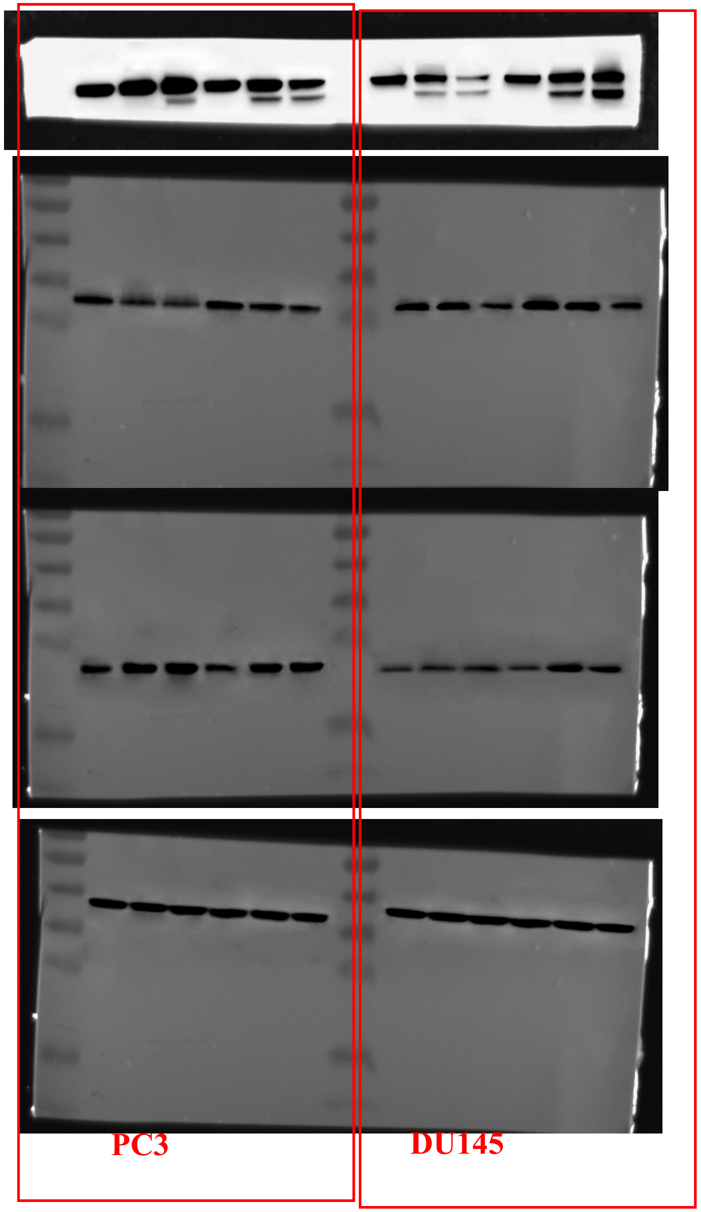

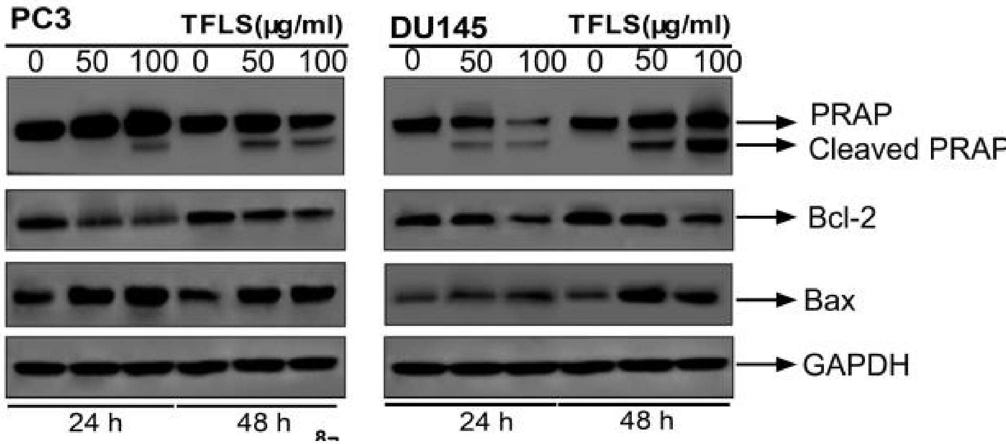

EMT marker in PC3 cells

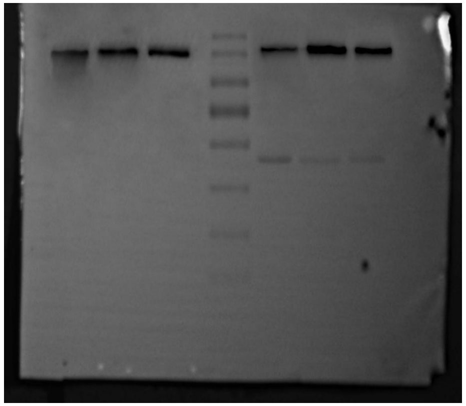

E-cadhernin

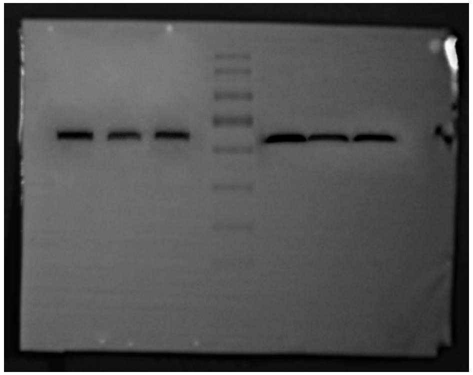

Vimentin

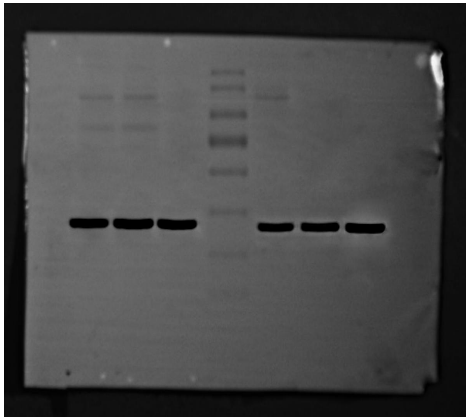

GAPDH

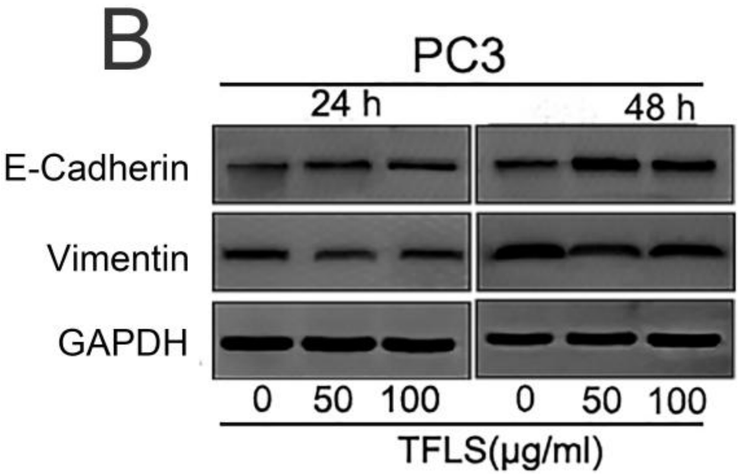

EMT marker in DU145 cells

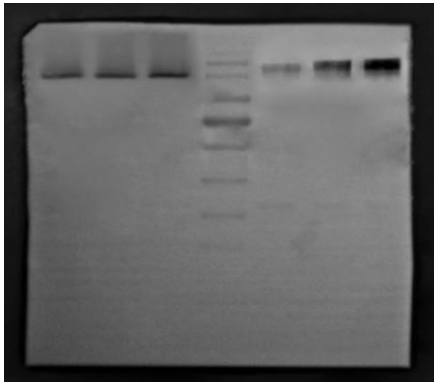

E-cadhernin

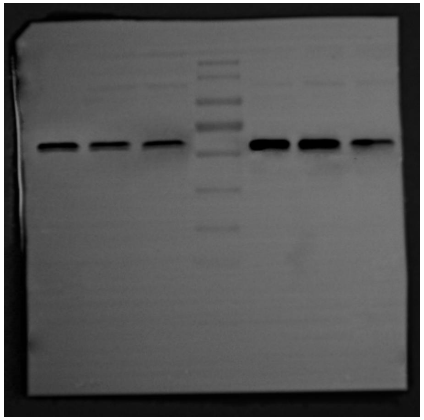

Vimentin

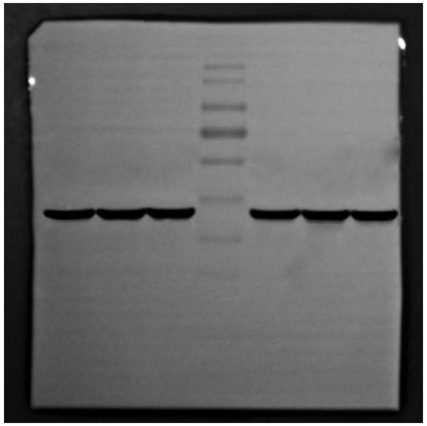

GAPDH

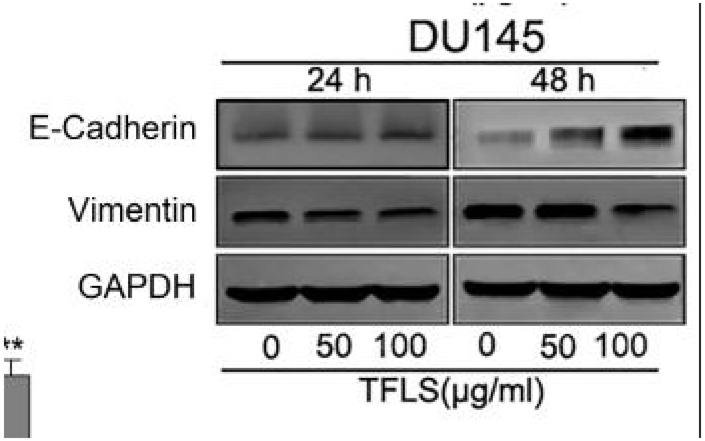

AKT pathway

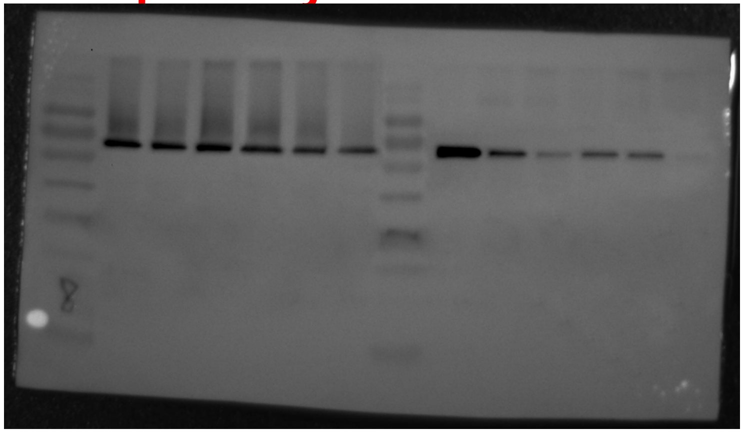

P-AKT

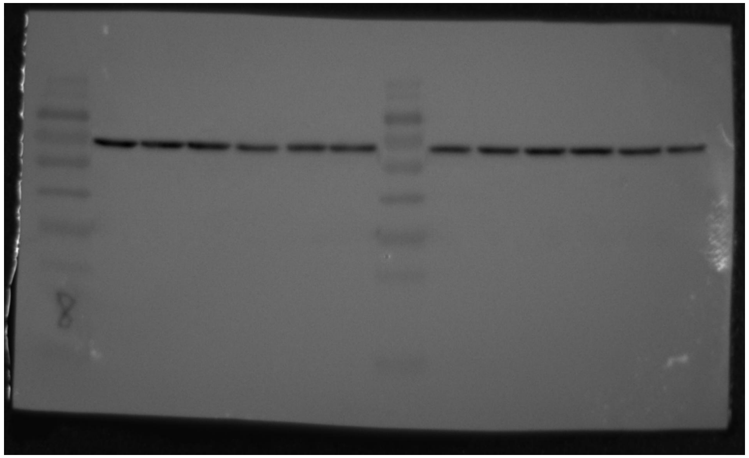

AKT

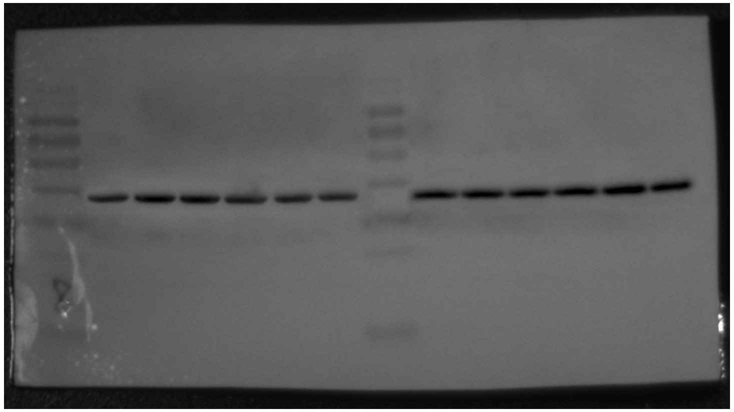

GAPDH

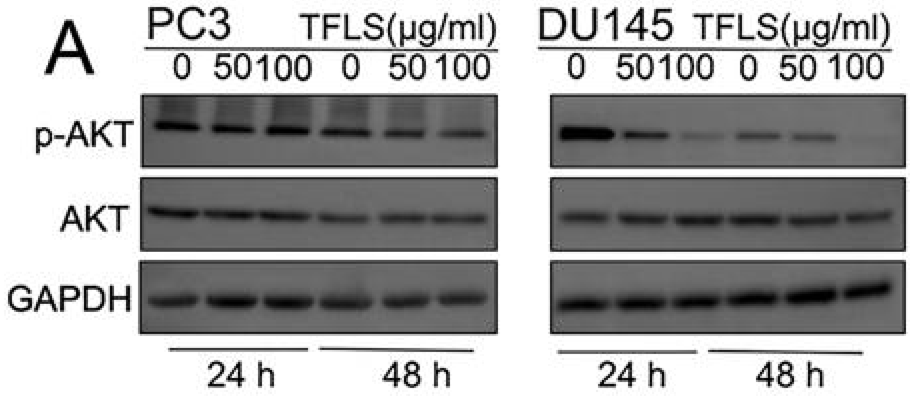

mTOR pathway

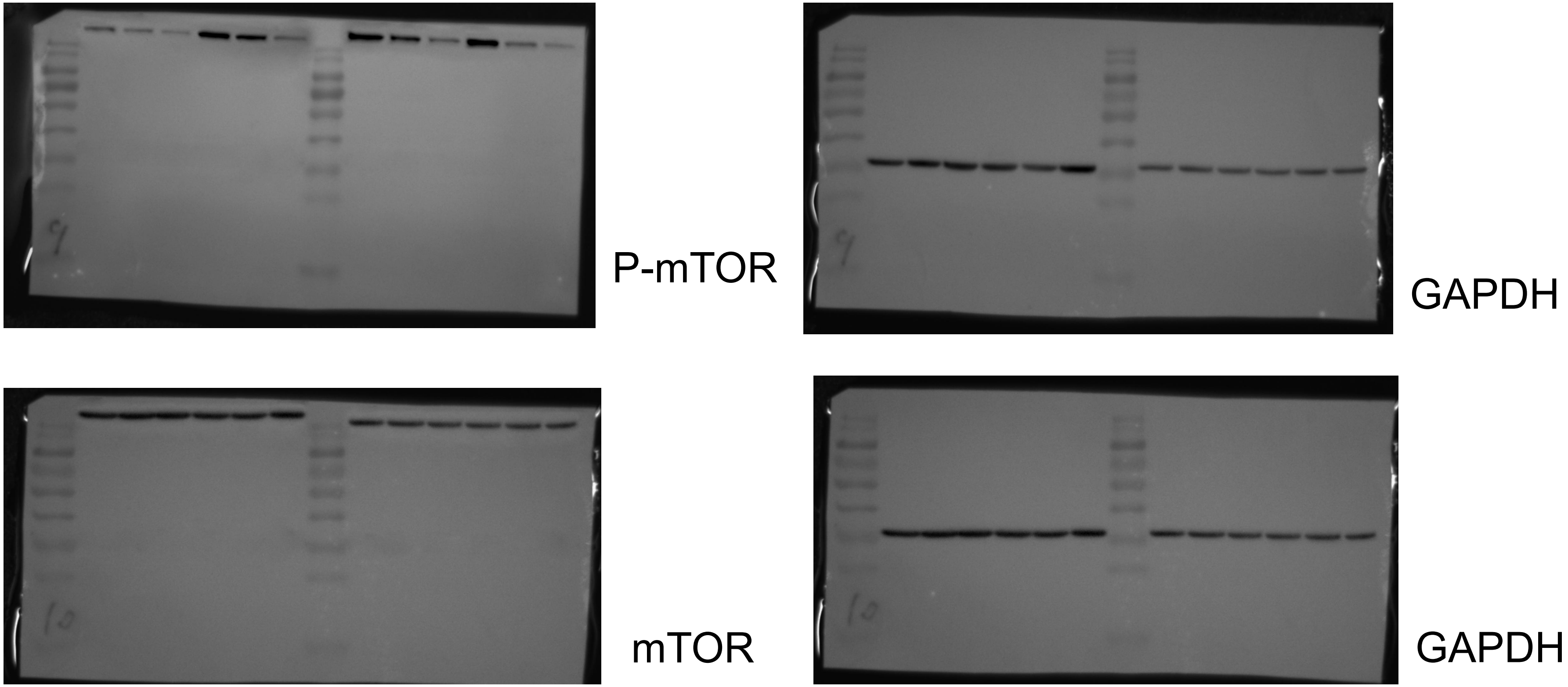

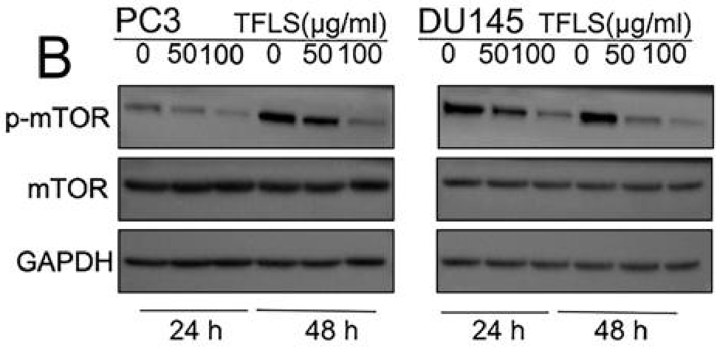

NF-kB pathway

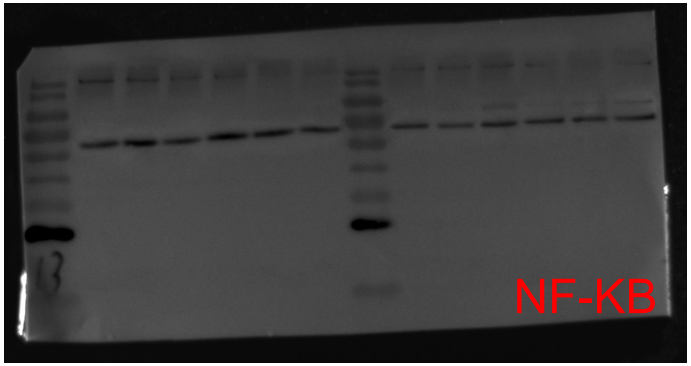

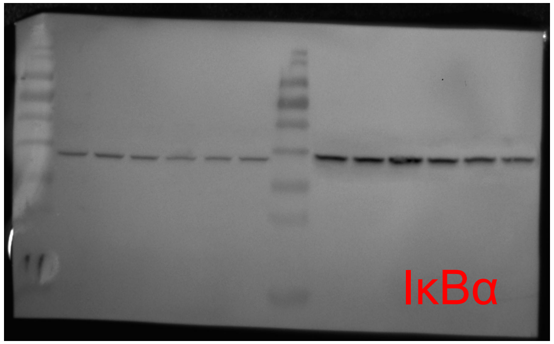

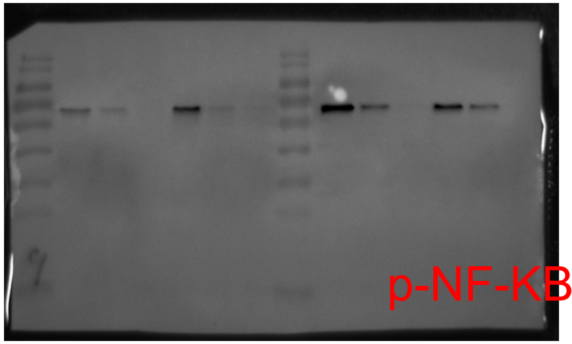

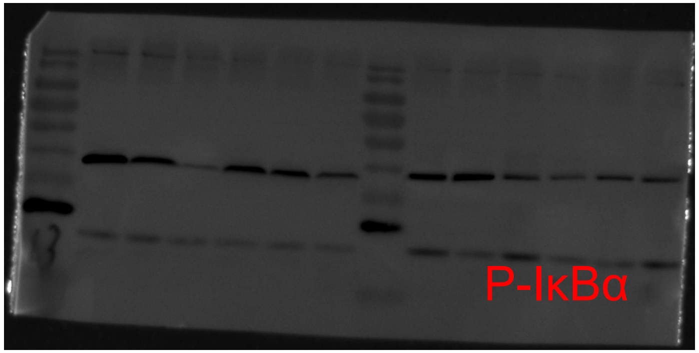

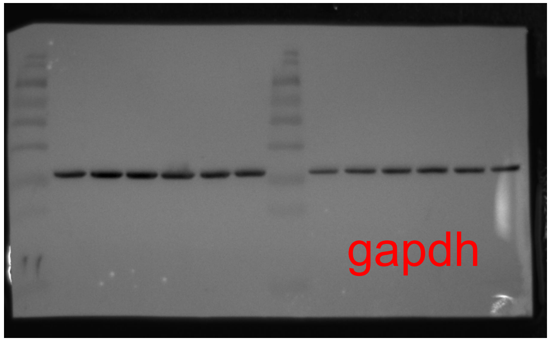

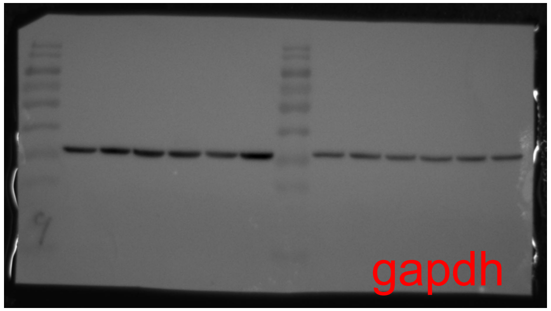

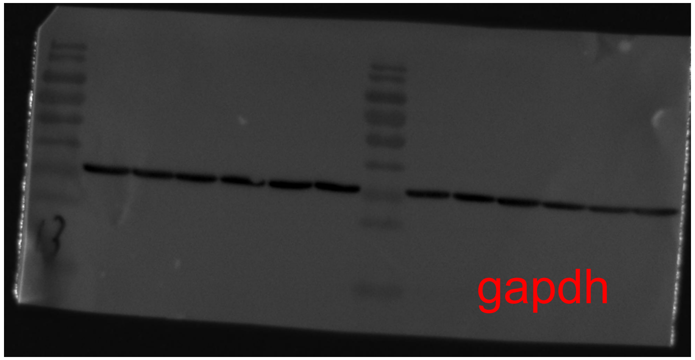

C

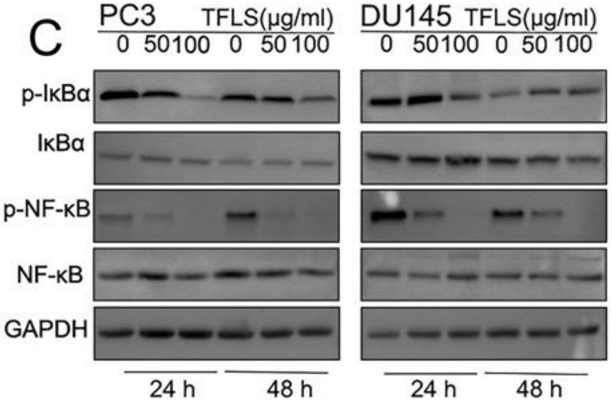

Supplement: Supplementary file 2 [file DataSheet1.PDF]
